# Joint trajectories of sleep duration and depressive symptoms and risk of incident multimorbidity: a longitudinal analysis with machine learning prediction

**DOI:** 10.1186/s12877-026-07694-2

**Published:** 2026-05-28

**Authors:** Jiecheng Jiang, Zhujiang Li, Zhuo Zhang, Shiyu Ji, Zefeng Zhang, Yixuan Wu, Yaqi Li, Mingyu Yu, Peipei Qiao, Junxiang Xu, Jun Wang, Panpan Huang

**Affiliations:** 1 School of Basic Medical Sciences, Hubei University of Chinese Medicine, Wu Han, 430065 China; 2 Engineering Research Center of TCM Protection Technology and New Product Development in Geriatric Brain Health, School of Basic Medicine, Ministry of Education, Hubei University of Chinese Medicine, Wu Han, 430065 China; 3 Hubei Shizhen Laboratory, Wu Han, 430065 China

**Keywords:** Sleep duration, Depressive symptoms, Joint trajectories, Chronic diseases, Multimorbidity, Machine learning, CHARLS

## Abstract

**Background:**

Sleep disturbances and depressive symptoms frequently co-occur in older adults. Both conditions follow distinct, time-varying trajectories. Nevertheless, most studies rely on cross-sectional assessments, limiting evidence regarding their joint longitudinal evolution and associations with incident chronic diseases.

**Methods:**

This study utilized data drawn from 3,221 participants (aged ≥ 60 years) enrolled in the China Health and Retirement Longitudinal Study (CHARLS). Group-based multi-trajectory models (GBMTM) were constructed using repeated measures from 2011 to 2018 to identify heterogeneous joint trajectories of sleep duration and depressive symptoms. Cox proportional hazards models assessed associations with 13 incident chronic diseases and multimorbidity. Additionally, a machine learning framework incorporating seven algorithms was applied to identify baseline predictors of high-risk trajectories, followed by SHAP analysis to enhance model interpretability.

**Results:**

The mean age of participants was 65.80 ± 4.93 years. We identified four joint trajectories: normal-stable sleep and low-stable depression (24.46%), short-stable sleep and low-stable depression (27.17%), normal-increasing sleep and moderate-increasing depression (25.00%), and short-decreasing sleep and high-increasing depression (23.38%). The “short-decreasing sleep and high-increasing depression” trajectory exhibited the highest risks, notably for memory-related disorders (HR = 3.08), stroke (HR = 2.56), and multimorbidity (HR = 1.97). XGBoost and ANN achieved the best predictive performance (AUC = 0.805), with body pain and cognitive function identified as primary predictors.

**Conclusion:**

The trajectory characterized by declining sleep duration and worsening depressive symptoms was associated with heightened risks of multimorbidity and various chronic conditions in older adults. These findings underscore the necessity of integrating sleep and depressive symptom surveillance for chronic disease prevention. Furthermore, early screening for body pain and cognitive decline may facilitate the timely identification of high-risk individuals and inform targeted precision interventions.

**Graphical Abstract:**

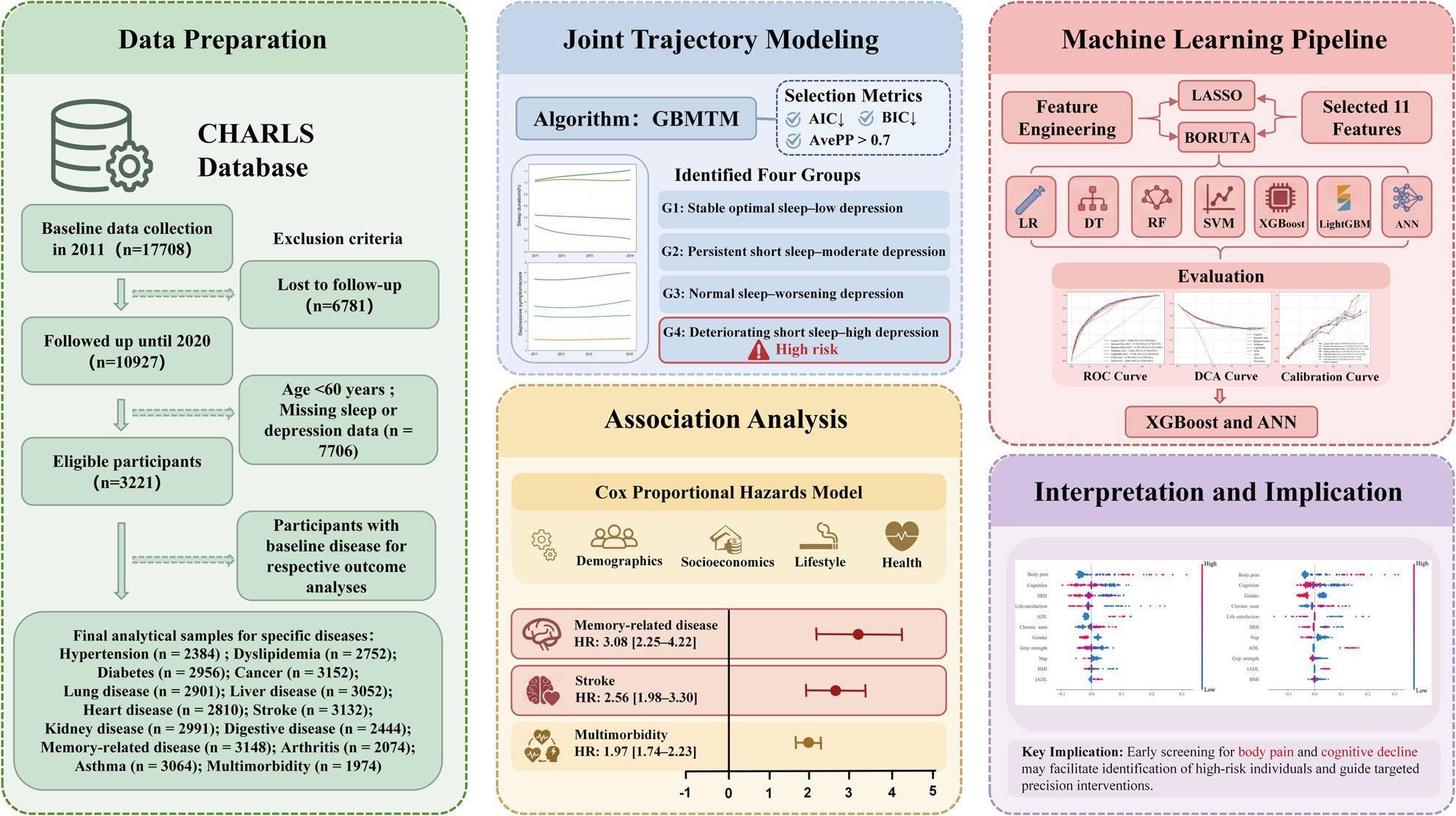

**Supplementary Information:**

The online version contains supplementary material available at 10.1186/s12877-026-07694-2.

## Introduction

The accelerating aging of the global population has intensified the impact of chronic non-communicable diseases (NCDs), representing a significant public health concern [1]. Sleep disturbances and depressive symptoms are highly prevalent and frequently comorbid among older adults [2, 3]. Epidemiological studies indicate that approximately 40% to 60% of individuals with depression experience concurrent sleep disturbances [4]. Conversely, persistent sleep problems significantly elevate the risk of developing depressive symptoms [5]. These interrelated conditions not only substantially impair quality of life in older adults but also act as independent predictors of multiple chronic diseases, such as cognitive decline, cardiovascular disease, and metabolic syndrome [6–9].

A reciprocal and intricate association links sleep patterns with depressive symptoms [10]. This interaction is likely driven by shared biological mechanisms, including hypothalamic-pituitary-adrenal (HPA) axis hyperactivation, autonomic nervous system dysregulation, and chronic systemic inflammation [11–13]. This pathophysiological interplay establishes a deleterious cycle that may synergistically accelerate physiological decline, thereby promoting the development of multiple chronic diseases [14–16]. Therefore, evaluating the joint progression of sleep disturbances and depression, rather than examining them in isolation, is essential to understand their cumulative adverse effects.

Although numerous investigations have examined the impact of sleep or depression on chronic diseases, the majority rely on cross-sectional designs that do not account for the temporal dynamics of these factors [17–19]. Moreover, existing research frequently examines sleep duration and depressive symptoms in isolation, overlooking their potential joint evolution over the life course [20, 21]. Given the dynamic nature of sleep patterns and depressive symptoms, relying on single-indicator trajectories may yield an incomplete assessment of an individual’s risk profile. Furthermore, nationally representative longitudinal studies linking joint sleep–depression trajectories to the incidence of multiple chronic diseases remain scarce.

To bridge this gap, we leveraged data from a nationally representative cohort, the China Health and Retirement Longitudinal Study (CHARLS). We applied group-based multi-trajectory modeling (GBMTM) to identify joint patterns of nighttime sleep duration and depressive symptoms in the aging population [22]. Subsequently, we examined the associations between these joint trajectories and the incidence of multiple chronic diseases. Additionally, we employed a machine learning framework to identify baseline predictors of high-risk trajectories. This study aims to characterize distinct high-risk subgroups and facilitate early screening, thereby informing targeted interventions for the prevention and management of chronic diseases in aging populations.

## Methods

### Study design and participants

The current study utilized data from the CHARLS, a continuous nationwide survey monitoring the aging process in China. Participants aged 45 years and above were recruited using a multistage probability-proportional-to-size (PPS) sampling method to ensure representativeness. The analysis spanned a ten-year period, including the 2011 baseline and four follow-up waves (2013, 2015, 2018, and 2020). Ethical approval for data collection was granted by the Peking University Institutional Review Board (No. IRB00001052-11015), and all respondents provided written informed consent prior to participation.

We analyzed data from five waves of the CHARLS cohort (2011–2020). Repeated measures from the first four waves (2011–2018) were used to construct joint trajectory models of sleep duration and depressive symptoms. From the 2011 baseline sample, we applied the following exclusion criteria: (1) age < 60 at baseline; (2) missing data on sleep duration or depressive symptoms at any time point during the modeling period. These exclusions resulted in a final analytical sample of 3,221 participants. The detailed participant selection process and the overall analytical framework of this study are illustrated in Figure S1.

### Measurement of sleep duration and depressive symptoms

Sleep duration was assessed based on self-reports, using consistent measurement protocols across all five waves. Participants reported their average actual nightly sleep duration during the preceding month.

The 10-item Center for Epidemiologic Studies Depression Scale (CES-D-10) was utilized to measure depressive symptoms. Each item was scored on a scale of 0 to 3 representing the frequency of symptoms. The aggregate score spans from 0 to 30, where elevated scores signify more severe depressive symptoms. The psychometric properties of this instrument have been validated in Chinese cohorts [23–25].

### Definition of incident chronic diseases and multimorbidity

The primary outcome was the incidence of 13 chronic conditions assessed during the 2011–2020 follow-up period. These conditions included dyslipidemia, diabetes, hypertension, heart disease, stroke, chronic lung disease, asthma, kidney disease, liver disease, digestive disease, cancer, arthritis, and memory-related disorders. Diagnoses of these conditions were ascertained through participants’ self-reports of physician diagnosis. Multimorbidity was defined as the coexistence of two or more of these chronic conditions. To identify incident cases, participants reporting a specific disease at baseline (2011) were excluded from the analysis of that specific condition. For the analysis of incident multimorbidity, participants with two or more chronic conditions at baseline were excluded. An incident event was defined as the first report of a diagnosis during the follow-up period.

### Covariates

We adjusted for baseline covariates across four domains based on prior literature to account for potential confounding: (1) Demographic characteristics: gender, age, education attainment, marital status, Hukou, residence, and geographic region; (2) Socioeconomic factors: retirement status, medical insurance coverage, household per capita consumption expenditure (HPCC), and life satisfaction; (3) Lifestyle factors: smoking status, alcohol consumption, nap duration, social participation, and social isolation score; (4) Health-related indicators: waist circumference, grip strength, body mass index (BMI), balance, chair stand test, blood pressure, lung function, activities of daily living (ADL/IADL), disability status, sensory function (vision/hearing), tooth loss, self-rated health (SRH), body pain, history of falls, cognitive score, and baseline chronic disease count. Detailed definitions and categorization criteria for these covariates are provided in Table S1.

### Statistical analysis

To capture the dynamic joint progression of sleep duration and depressive symptoms, we employed GBMTM using the ‘gbmt’ package in R [26, 27]. We evaluated models ranging from 2 to 6 latent classes, including polynomial terms to accommodate nonlinear trends. The optimal model structure was determined by balancing statistical performance and interpretability, adhering to the following criteria: (1) minimization of both the Bayesian Information Criterion (BIC) and Akaike Information Criterion (AIC); (2) robust classification precision, evidenced by an average posterior probability (AvePP) exceeding 0.7 per class; and (3) a requirement that each trajectory group comprise at least 5% of the study population. The trajectory groups were labeled using a “level-trend” naming framework [28, 29], which integrates the overall clinical level of the trajectory with its longitudinal mathematical trend. Specifically, the revised labels were constructed from two dimensions: ① Clinical Level: defined according to the overall level of the trajectory relative to established epidemiological cutoffs (Sleep: Short < 6 h, Normal 6–8 h, Long > 8 h [30]; Depression: Low ≤ 9, Moderate 10–14, High ≥ 15 [31]); ② Mathematical Trend: described according to the longitudinal slope of the trajectory (Stable, Increasing, or Decreasing). Cox proportional hazards regression models were used to estimate the associations between joint trajectory groups and the risk of incident chronic diseases and multimorbidity. We checked the proportional hazards assumption using the Schoenfeld residuals method [32]. We adopted a hierarchical adjustment strategy: Model 1 adjusted for demographic characteristics; Model 2 additionally incorporated socioeconomic status and lifestyle behaviors; Model 3 further controlled for health-related indicators. Results are expressed as hazard ratios (HRs) accompanied by 95% confidence intervals (CIs). To address missing baseline covariates (< 20%), we applied the multiple imputation by chained equations (MICE) technique. Cumulative incidence rates were visualized using Kaplan–Meier curves, and discrepancies among groups were evaluated using log-rank tests.

To identify key predictors of membership in the high-risk trajectory, we implemented a machine learning pipeline. This analysis was designed to identify the most informative baseline predictors of high-risk trajectory membership and to support individualized risk prediction. The dataset was randomly divided into training and testing sets at a ratio of 7:3. Optimal baseline features were isolated using a dual-stage selection process that integrated the Boruta algorithm with LASSO regression [33]. We compared the predictive performance of seven algorithms: Artificial Neural Network (ANN), LightGBM, XGBoost, Support Vector Machine (SVM), Random Forest (RF), Decision Tree (DT), and Logistic Regression (LR). Hyperparameters were optimized using a 10-fold cross-validated grid search on the training set [34]. We assessed predictive capability primarily via the area under the receiver operating characteristic curve (AUC), supplemented by metrics including specificity, sensitivity, F1 score, and accuracy. Decision Curve Analysis (DCA) was used to assess clinical utility, while calibration plots served to inspect the alignment of predicted risks with observed events. We employed the SHapley Additive exPlanations (SHAP) analysis to improve the interpretability of the optimal model. SHAP summary plots visualized global feature importance and directional effects, while force plots illustrated individual-level risk attribution [35]. To facilitate individualized risk assessment, the optimal machine learning model was deployed as an interactive, open-access web application.

Two distinct sensitivity analyses were performed to verify the stability of our results. First, we repeated the primary analyses using unimputed data to evaluate potential bias from missing data imputation. Second, to reduce temporal ambiguity and minimize potential reverse causality, we excluded participants with chronic diseases diagnosed prior to 2018. We then examined the associations between joint trajectories and incident chronic diseases during the 2018–2020 follow-up.

Statistical significance was defined as a two-sided P value < 0.05. The statistical packages R (version 4.4.2) and Python (version 3.11.7) were utilized for all data computations.

## Results

### Identification of joint trajectories and participant characteristics

**Fig. 1:**
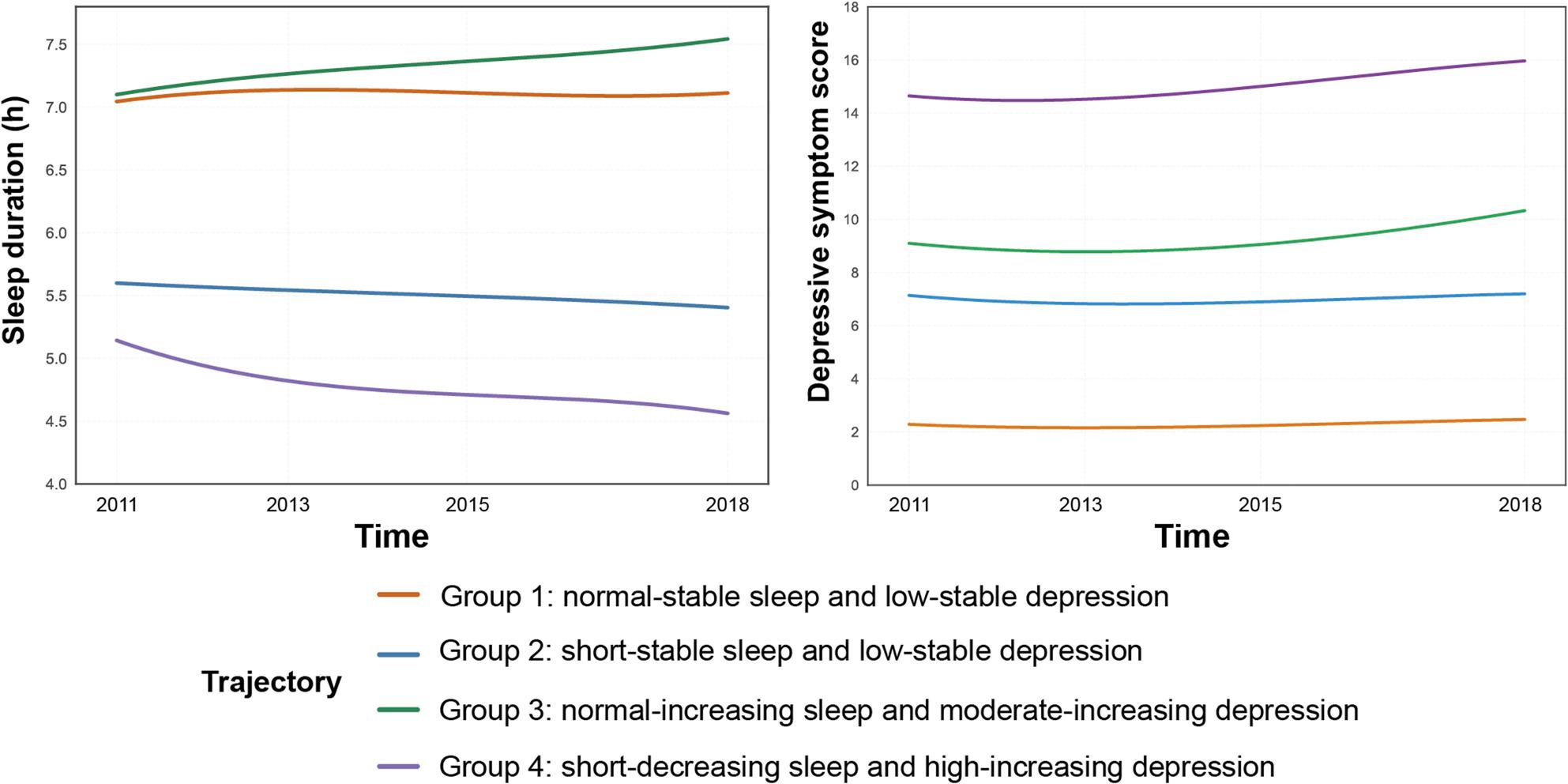
Joint longitudinal trajectories of nighttime sleep duration and depressive symptoms (2011–2018)

The final analytical sample comprised 3,221 individuals, with an average age of 65.80 ± 4.93 years. Based on model fit statistics and classification criteria (Table S2), four distinct joint trajectories were identified (Fig. 1). Group 1, “normal-stable sleep and low-stable depression” (*n* = 788; 24.46%), served as the reference, characterized by stable sleep duration (~ 7 h) and low depressive symptoms. Group 2, “short-stable sleep and low-stable depression” (*n* = 875; 27.17%), exhibited consistently short sleep duration and low depressive symptoms. Group 3, “normal-increasing sleep and moderate-increasing depression” (*n* = 805; 25.00%), exhibited normal but gradually increasing sleep duration alongside markedly increasing depressive symptoms over time. Finally, Group 4, “short-decreasing sleep and high-increasing depression” (*n* = 753; 23.38%), showed a pronounced decline in sleep duration accompanied by severe, progressively increasing depressive symptoms.

Baseline demographic and clinical characteristics stratified by joint trajectory group are summarized in Table S3. Significant between-group differences were observed for all covariates (*P* < 0.001), except age and blood pressure (systolic and diastolic). Compared with other groups, participants in Group 4 were more likely to be female, reside in rural areas, and have lower educational attainment and household consumption. Regarding health status, this group exhibited lower grip strength, more severe bodily pain, poorer cognitive function, and a higher prevalence of multimorbidity.

### Longitudinal associations between joint trajectories and incident chronic diseases and multimorbidity

**Fig. 2:**
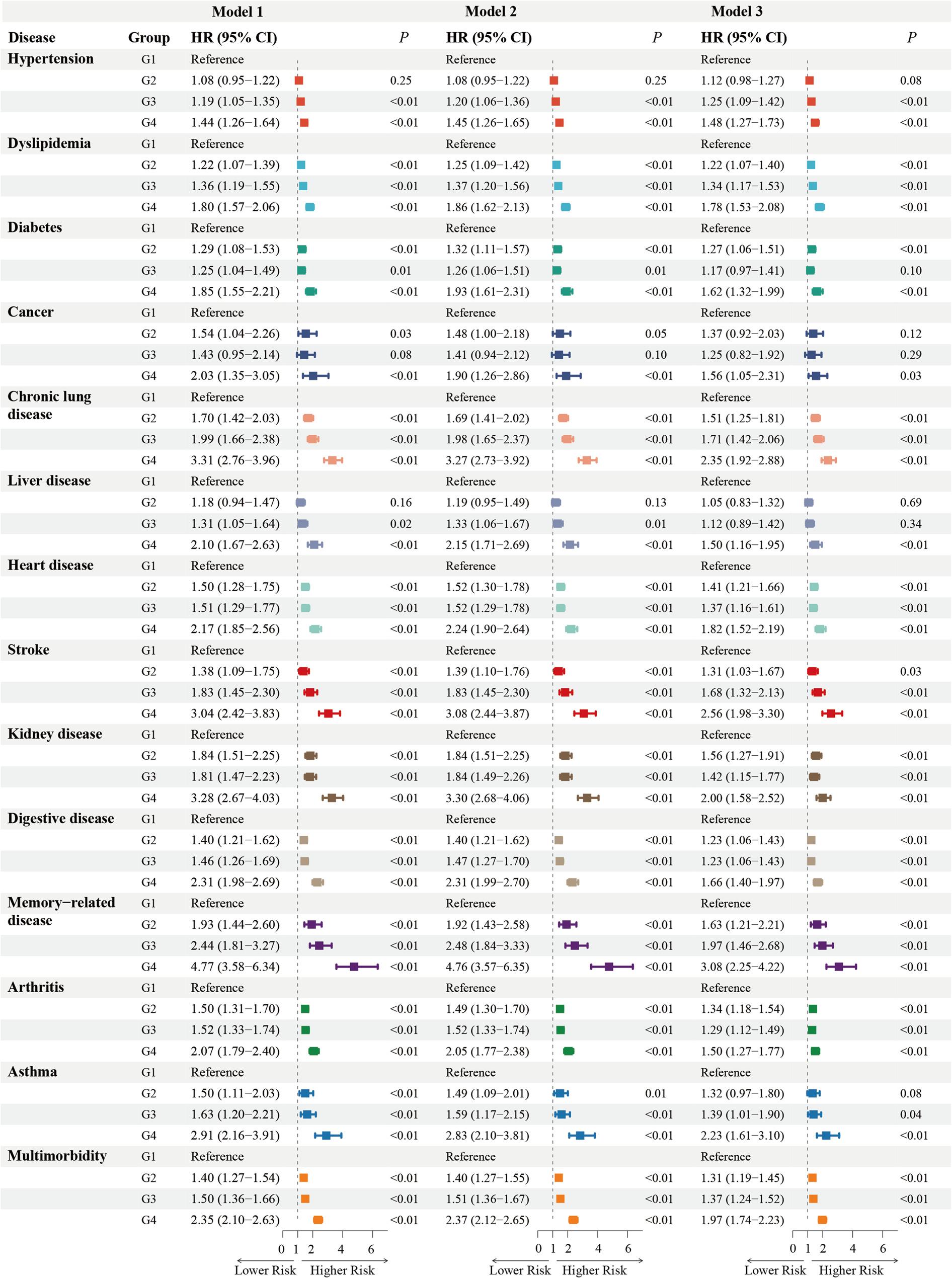
Forest plot of joint sleep–depression trajectories and risk of multiple incident chronic diseases and multimorbidity

Cox proportional hazards regression analysis revealed independent associations between joint sleep–depression trajectories and the risk of multiple incident chronic diseases (Fig. 2, Table S4). In the fully adjusted model, Group 4 exhibited consistently elevated risks for all assessed conditions compared with Group 1. Specifically, participants in Group 4 had the highest risk for memory-related disorders (HR = 3.08, 95% CI: 2.25–4.22). The risk more than doubled for stroke (HR = 2.56, 95% CI: 1.98–3.30), chronic lung disease (HR = 2.35, 95% CI: 1.92–2.88), asthma (HR = 2.23, 95% CI: 1.61–3.10), and kidney disease (HR = 2.00, 95% CI: 1.58–2.52). Furthermore, significant risk increases were observed for heart disease (HR = 1.82, 95% CI: 1.52–2.19), dyslipidemia (HR = 1.78, 95% CI: 1.53–2.08), digestive disease (HR = 1.66, 95% CI: 1.40–1.97), and diabetes (HR = 1.63, 95% CI: 1.32–1.99). Elevated risks were also noted for cancer, liver disease, arthritis, and hypertension, with HRs ranging from 1.48 to 1.56 (all *P* < 0.05). Regarding overall disease burden, Group 4 exhibited a 97% higher risk of multimorbidity (HR = 1.97, 95% CI: 1.74–2.23). To further evaluate heterogeneity across trajectory groups, we conducted additional pairwise comparisons in the fully adjusted Cox models, with Group 2 serving as the reference category (Table S10). These analyses showed that Group 3 had significantly higher risks of memory-related disorders and stroke than Group 2, whereas no statistically significant differences were observed for most other incident chronic diseases and multimorbidity.

**Fig. 3:**
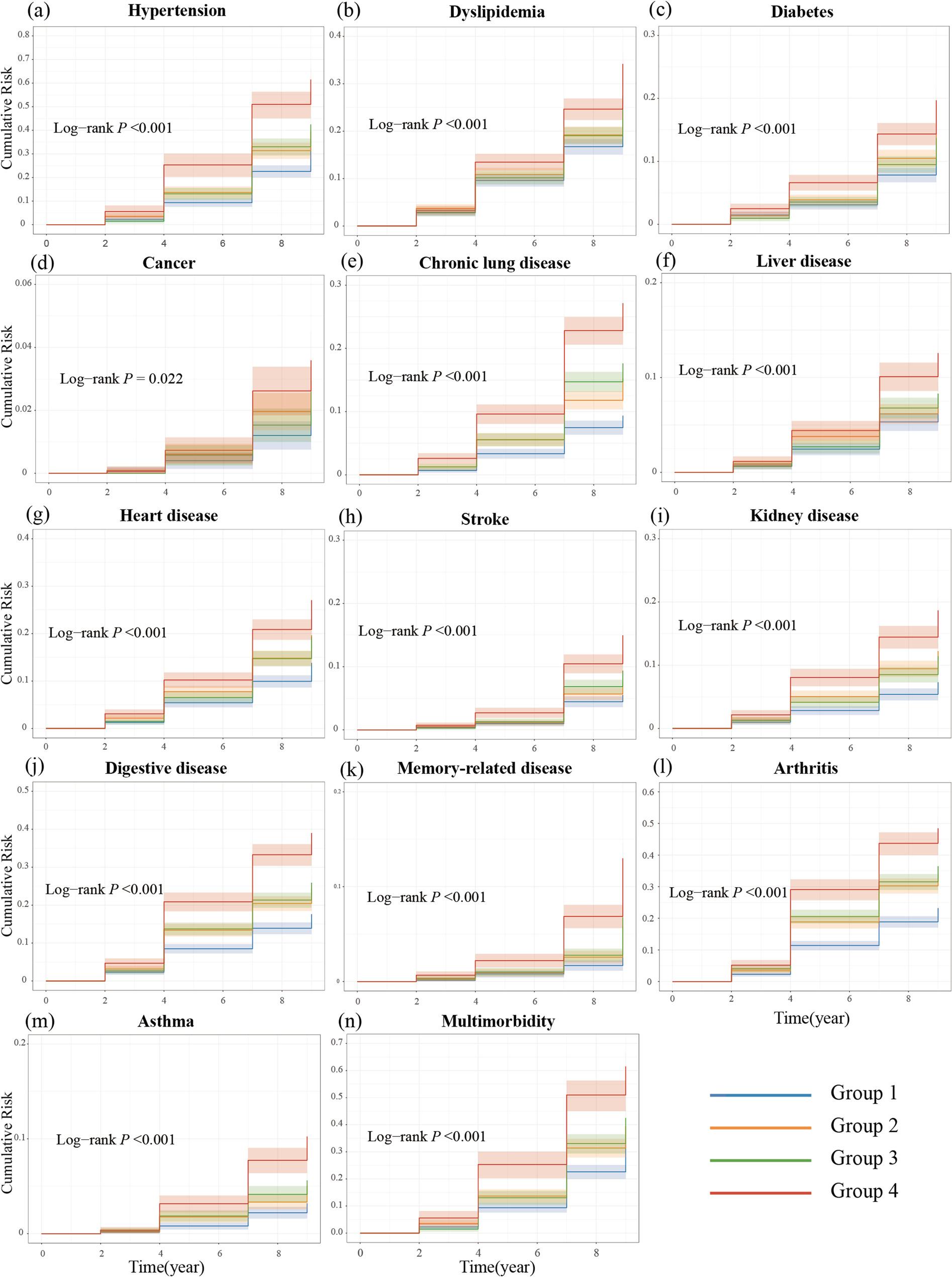
Kaplan–Meier cumulative incidence curves for chronic diseases and multimorbidity stratified by joint trajectory groups. **a**–**m** Cumulative incidence curves for the 13 specific chronic diseases; **n** cumulative incidence curve for multimorbidity

Kaplan–Meier survival curves (Fig. 3) illustrate the cumulative incidence of all evaluated outcomes by joint trajectory groups. Log-rank tests confirmed significant differences in time-to-event distributions among groups for all outcomes ( *P* < 0.001). Consistent with the Cox regression findings, Group 4 showed the steepest increase in cumulative incidence across all outcomes, indicating a more rapid accumulation of disease burden compared with other groups.

### Identification of key predictive features and model interpretation

**Fig. 4:**
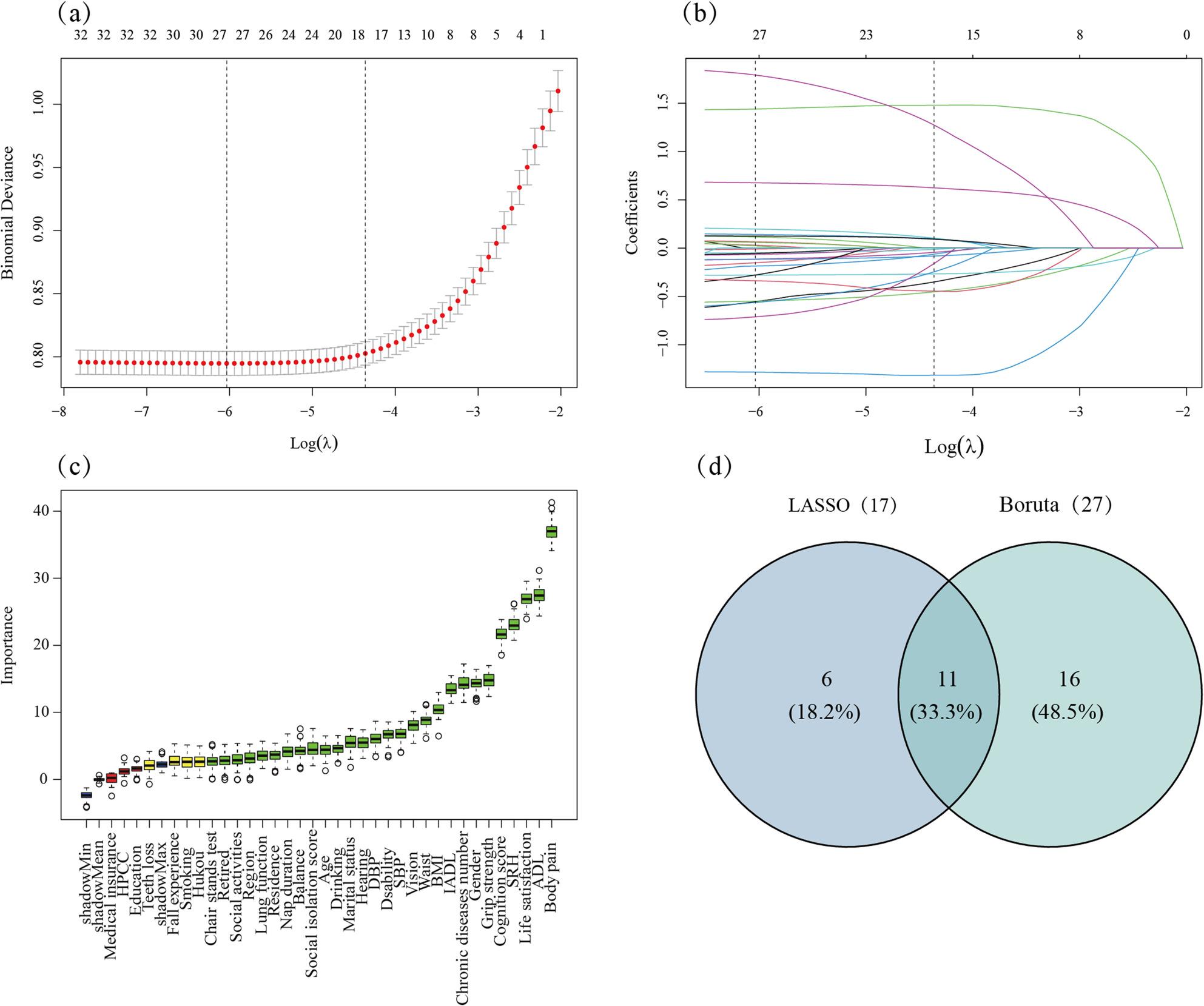
Feature selection analysis results. **a** LASSO parameter selection via cross-validation; **b** LASSO coefficient profiles: The left dashed line indicates the minimum error(λ_min_), while the right dashed line indicates the 1-standard-error rule(λ_1se_); **c** Feature importance ranking via the Boruta algorithm: green boxplots indicate confirmed important features, and blue boxplots represent shadow features; **d** Venn diagram illustrating the intersection of selected features

Through 10-fold cross-validation, the ideal regularization parameter log(λ) was determined, retaining 17 predictors with non-zero coefficients (Figs. 4a–b). Concurrently, the Boruta method identified 27 confirmed attributes (Fig. 4c). The intersection of these two selection methods identified a final set of 11 key features (Fig. 4d), which were used as inputs for the prediction models.

**Fig. 5:**
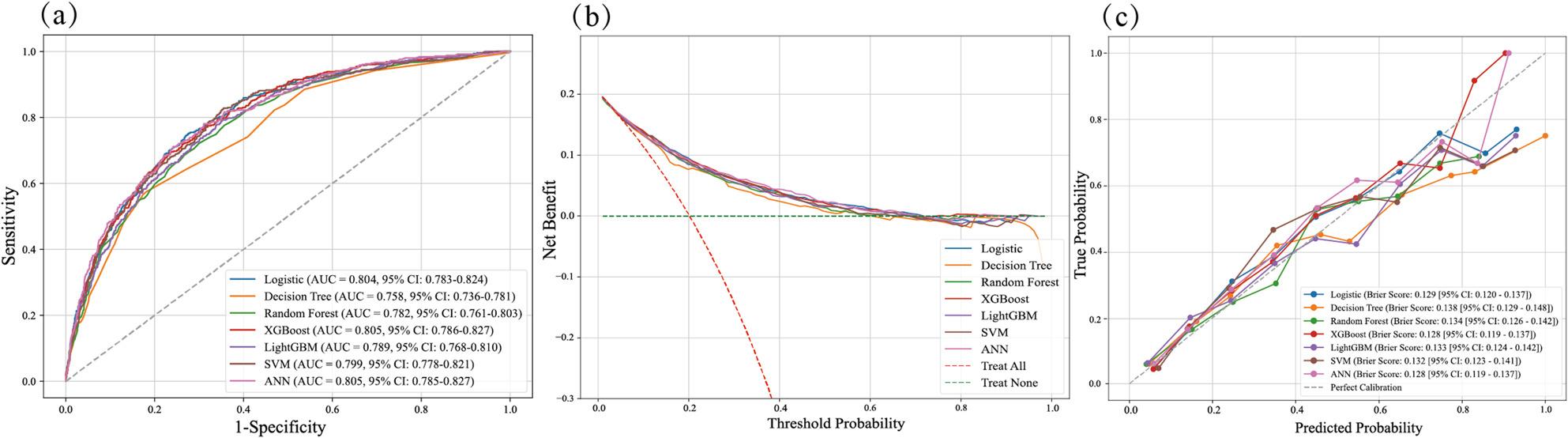
Comparative performance of seven predictive models in the test set. **a** ROC curves; **b** decision curve analysis (DCA); **c** calibration curves

Using these key features, we evaluated the performance of seven machine learning algorithms on the test set. ROC curve analysis (Fig. 5a) indicated that XGBoost and ANN achieved comparably superior performance, both yielding an AUC of 0.805 (95% CI: 0.785–0.827). Additionally, these models outperformed the other five algorithms in accuracy, sensitivity, and F1 score (Table S5). Decision curve analysis (DCA, Fig. 5b) demonstrated that both XGBoost and ANN provided substantially greater net benefit compared with “treat-all” or “treat-none” strategies. Moreover, calibration curves (Fig. 5c) showed strong agreement between predicted and observed probabilities, indicating excellent calibration. Together, these performance evaluations suggest that the selected baseline features can jointly provide reasonably accurate individualized risk prediction and may support early risk stratification.

**Fig. 6:**
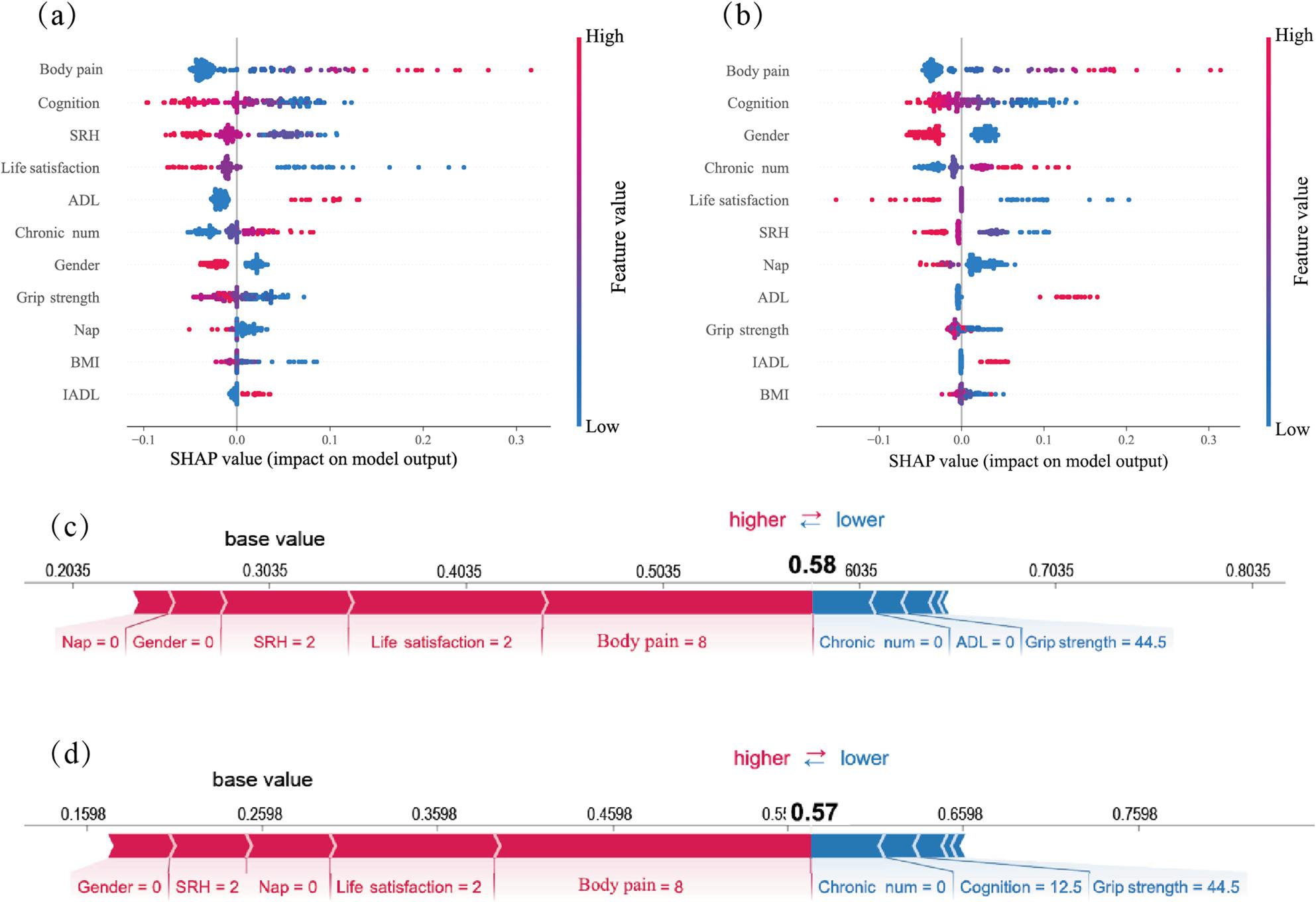
SHAP-based model interpretability. **a**–**b** SHAP beeswarm plots illustrating global feature importance for XGBoost and ANN models. The x-axis represents the SHAP value, with each row corresponding to a feature. Red and blue dots indicate high and low feature values, respectively; **c**–**d** local interpretability analysis for two representative individual cases

Global and local interpretability of the top-performing models (ANN and XGBoost) was achieved using the SHAP method. SHAP summary plots (Figs. 6a–b) ranked features by mean absolute SHAP values, illustrating global importance. Baseline body pain emerged as the most influential predictor of high-risk trajectory membership, followed by cognitive function, self-rated health, and life satisfaction. Directional analysis revealed that higher levels of body pain, impaired ADL, and a greater number of chronic conditions were associated with elevated SHAP values, indicating an increased probability of classification into the high-risk group. Conversely, better cognitive function, life satisfaction, grip strength, and self-rated health were associated with lower SHAP values, suggesting protective effects. Finally, individual SHAP force plots (Figs. 6c–d) visualized feature contributions for specific participants, providing detailed insights into individual risk profiles. Based on the finalized model, we developed a machine learning-driven web application (Figure S8), accessible at https://sleep-depression-risk-app.streamlit.app. By inputting an individual’s baseline characteristics, the online tool automatically estimates the probability of belonging to the high-risk trajectory, thereby supporting early risk stratification and preventive attention.

### Sensitivity analysis

Sensitivity analyses confirmed the robustness of our results. Analyses using unimputed data (Table S6) replicated the primary findings in both Cox regression (Table S7, Figure S2) and Kaplan–Meier survival assessments (Figure S3). Furthermore, machine learning validation remained consistent (Table S8); XGBoost demonstrated optimal performance, while SHAP analysis identified body pain and cognitive scores as the primary predictors (Figure S4–6). Similarly, in sensitivity analyses excluding participants with chronic conditions prior to 2018, the “short-decreasing sleep and high-increasing depression” trajectory remained significantly associated with incident multimorbidity and most specific diseases (Table S9, Figure S7).

## Discussion

Leveraging the nationally representative CHARLS longitudinal cohort, we applied GBMTM to characterize the joint progression of depressive symptoms and sleep duration in the aging Chinese population. In contrast to prior research focusing on static, single-indicator associations, our analysis identified four distinct joint trajectory patterns. Importantly, these trajectory groups should be understood as an empirical summary of the major joint longitudinal patterns observed in this cohort, rather than as a set of validated clinical phenotypes. From a descriptive perspective, they may still provide clinically relevant pattern distinctions by differentiating relatively stable profiles from those characterized by persistent short sleep, worsening depressive symptoms, or concurrent deterioration in both domains [28]. At the same time, as with other latent class approaches, some theoretically conceivable patterns may not emerge as separate groups if they are not sufficiently prevalent or distinct within the study population, and some within-group heterogeneity is likely to remain [36, 37].

Approximately 23% of participants were classified into the “short-decreasing sleep and high-increasing depression” trajectory (Group 4). This trajectory represents a particularly deleterious phenotype, characterized by a sharp decline in sleep duration and progressively worsening depressive symptoms. Cox regression analyses revealed that individuals in this group faced significantly elevated risks for all 13 assessed incident chronic diseases and multimorbidity. Notably, the risk magnitude was most pronounced for memory-related disorders and stroke, followed by chronic lung disease, asthma, and kidney disease. By integrating machine learning with SHAP analysis, we identified body pain and cognitive function as primary predictors of this high-risk profile.

Previous research has independently linked sleep disturbances and depressive symptoms to a range of chronic conditions. Longitudinal studies indicate that sleep disturbances elevate susceptibility to hypertension, coronary heart disease, and heart failure, likely mediated by sympathetic activation and endothelial dysfunction [6, 38, 39]. Additionally, sleep disruption impairs glucose metabolism and appetite regulation, promoting insulin resistance and type 2 diabetes [40]. Similarly, depressive symptoms have consistently been recognized as independent predictors of cardiovascular and metabolic dysfunctions [41, 42]. Building on these established independent associations, our study elucidates the dynamic and cumulative burden of their joint progression. We observed a distinct risk gradient and specific disease vulnerability profiles across the trajectory groups. Additional pairwise comparisons showed that, while Groups 2 and 3 shared similar risk patterns for most chronic diseases, Group 3 had significantly higher risks of memory-related disorders and stroke than Group 2. This pattern suggests that Group 3 may carry a relatively greater risk of memory-related and cerebrovascular outcomes than Group 2. For other outcomes, however, the differences between Groups 2 and 3 should be interpreted with caution. Notably, Group 4, characterized by concurrent sleep deterioration and severe depressive symptoms, exhibited the highest aggregate risk across all examined health outcomes. These findings align with the allostatic load theory [43], suggesting that sleep disturbances and depression act synergistically rather than as isolated stressors, with potential implications for physiological dysregulation and systemic deterioration. We propose that this synergy may reflect a positive feedback loop between disrupted sleep regulation and depressive symptoms [44, 45]. Specifically, sleep fragmentation and circadian dysregulation may impair prefrontal cortex function and HPA axis negative feedback, thereby compromising stress resilience. In turn, persistent emotional disturbances sustain HPA axis hyperactivation, further exacerbating sleep disruption [46]. This bidirectional interaction may be linked to the accumulation of allostatic load, depletion of physiological reserves and multisystem health deterioration.

The elevated hazard ratios for memory-related disorders (HR = 3.08) and stroke (HR = 2.56) indicate that the “short-decreasing sleep and high-increasing depression” trajectory may be associated with impaired neurovascular integrity. Beyond established theories of HPA axis hyperactivation [47–49], recent evidence implicates a convergence of bioenergetic failure and immunometabolic dysregulation. Mechanistically, fragmented sleep is thought to disrupt aquaporin-4 (AQP4) polarization on astrocytic endfeet. This disruption impairs glymphatic clearance, promoting the aggregation of neurotoxic proteins notably amyloid-beta (Aβ) and tau [38, 50, 51]. Furthermore, astrocyte-neuron metabolic uncoupling potentially exacerbates this pathology. This process triggers mitochondrial stress-induced cGAS-STING activation, propagating sterile inflammation. Subsequently, this inflammatory environment may induce a pro-inflammatory microglial phenotype, initiating complement-mediated synaptic pruning and disrupting memory consolidation networks [52–55]. Concurrently, inflammation and oxidative stress facilitate MMP-9-mediated pericyte detachment and endothelial dysfunction, potentially leading to cerebral capillary rarefaction [56, 57]. The interplay between synaptic loss and microvascular degeneration likely creates a “vulnerable brain” state, thereby lowering the threshold for ischemic events.

Notably, the substantial risks observed for respiratory diseases (chronic lung disease and asthma) and kidney disease imply broader systemic dysregulation. We propose that mitochondrial dysfunction and oxidative stress may be involved in this multisystem decline. Emerging research indicates that both circadian misalignment and depressive states impair mitochondrial bioenergetics, leading to excessive generation of reactive oxygen species (ROS) [58, 59]. In highly vascularized organs susceptible to oxidative damage, such as the lungs and kidneys, this oxidative stress accelerates cellular senescence and tissue fibrosis [60, 61]. Furthermore, the gut-brain axis probably serves a crucial mediating function. Chronic sleep disruption and emotional stress induce gut dysbiosis, which enhances intestinal permeability and promotes the translocation of bacterial endotoxins (LPS) into the circulation. This low-grade endotoxemia may be linked to a systemic inflammatory cascade extending to distal organs, potentially exacerbating airway inflammation and renal microvascular damage [62–66]. Ultimately, the convergence of neurovascular unit dysfunction, mitochondrial exhaustion, and dysbiosis-driven systemic inflammation may help explain the extensive multimorbidity observed in the high-risk trajectory.

While our trajectory analyses quantified the long-term health risks associated with concurrent sleep deterioration and worsening depressive symptoms, they also underscore the need to identify individuals who are most likely to follow this high-risk trajectory at an early stage. In this context, the machine learning analysis identified body pain and cognitive function as the most informative baseline indicators of high-risk trajectory membership. Clinically, pain, sleep disturbance, and depressive symptoms frequently co-occur in older adults and may reinforce one another over time [67, 68]. Accordingly, body pain may serve as a readily observable marker of concurrent deterioration in sleep and depressive symptoms, particularly when accompanied by poorer cognitive performance or unfavorable self-rated health. These findings suggest that greater clinical attention to pain complaints and cognitive decline in older adults may help improve early recognition of high-risk individuals, while timely pain management combined with routine cognitive monitoring may offer a practical strategy for early intervention. To further support this process in practice, we deployed our optimal machine learning model as a user-friendly web-based risk calculator (https://sleep-depression-risk-app.streamlit.app). By integrating the selected key predictors, this tool provides a practical means of estimating individualized risk probabilities and may assist early risk stratification and targeted preventive attention in older adults.

Several limitations of this study warrant acknowledgment. First, reliance on self-reported depressive symptoms and sleep duration may result in recall bias. While the CES-D is a validated screening instrument, it assesses symptom severity rather than establishing a clinical diagnosis, which may lead to misclassification. Second, defining incident chronic diseases via self-reported physician diagnoses may introduce recall bias and misclassification, which could affect outcome ascertainment and bias the estimated associations. However, validation studies within the CHARLS cohort have shown acceptable concordance between self-reported diagnoses and objective medical data for some major conditions [69], partly supporting the reliability of our outcome assessment. Third, our sample consisted exclusively of Chinese individuals aged 60 and above. Differing cultural norms, healthcare systems, and socioeconomic contexts may influence the specific prevalence and patterns of the identified trajectories. Therefore, caution should be exercised when generalizing these findings to other populations, and future studies across diverse cultural and socioeconomic settings are needed to confirm external validity. Fourth, despite rigorous adjustment for covariates, residual confounding from unmeasured factors cannot be fully excluded. In addition, some variables included in the fully adjusted model may have contributed to overadjustment, which could have influenced the estimated associations. Fifth, the overlap between the trajectory modeling period and disease ascertainment in the primary analysis—intended to maximize data utilization—may introduce temporal ambiguity and the possibility of reverse causality. Although sensitivity analyses restricted to the 2018–2020 window supported the robustness of the observed associations, the overlapping time windows in the main analysis warrant cautious interpretation regarding causality. Sixth, to prioritize early clinical screening, our machine learning analysis was intentionally restricted to a binary classification of the highest-risk trajectory. Consequently, this framework precludes the adequate characterization of scientifically meaningful heterogeneity among intermediate-risk profiles, particularly the distinction between Group 2 and Group 3. Given their divergent longitudinal pathways and differential disease vulnerability profiles, future studies should employ multiclass classification algorithms or trajectory-specific predictive modeling to precisely disentangle the baseline predictors driving these nuanced trajectory divergences. Seventh, the trajectory groups derived from GBMTM are data-driven, empirical latent classifications rather than externally validated clinical phenotypes. Consequently, they may not capture all theoretically possible longitudinal patterns, and some within-group heterogeneity may remain.

## Conclusion

Leveraging nationally representative cohort data, this study characterized distinct joint trajectories of sleep duration and depressive symptoms. Individuals within the “short-decreasing sleep and high-increasing depression” trajectory exhibited elevated risks of multiple chronic conditions and multimorbidity. These findings suggest that the synergistic interaction between sleep disturbances and depressive symptoms may be associated with the development of multimorbidity in older adults. Machine learning analysis identified body pain and cognitive function as key predictors of this high-risk phenotype, underscoring their utility for early risk stratification and targeted intervention. Future clinical strategies should prioritize integrated approaches combining sleep management, depressive symptom assessment, and pain mitigation to alleviate disease burden in aging populations.

## Supplementary Information

Supplementary Material.

## Data Availability

Data are publicly available at the CHARLS repository (https://charls.pku.edu.cn/).
