# Supplementary material for: Joint trajectories of sleep duration and depressive symptoms and risk of incident multimorbidity: a longitudinal analysis with machine learning prediction
Source: BMC Geriatr. 2026 May 28;26:983. doi: 10.1186/s12877-026-07694-2 (PMC13411102; doi:10.1186/s12877-026-07694-2)
Supplement: Supplementary file 1 — Supplementary Material. [file 12877_2026_7694_MOESM1_ESM.docx]

**Supplementary materials**

**Table S1.** Definitions and measurement criteria for key study variables.

**Table S2.** Goodness-of-fit statistics for joint trajectory models with 1 to 6 latent classes.

**Table S3.** Baseline characteristics of participants.

**Table S4.** Cox proportional hazards regression analysis of the association between joint sleep–depression trajectories and the risk of incident chronic diseases and multimorbidity.

**Table S5.** Comparative performance metrics of seven machine learning algorithms in the validation set.

**Table S6.** Baseline characteristics of participants (unimputed data).

**Table S7.** Cox proportional hazards regression analysis of the association between joint sleep–depression trajectories and the risk of incident chronic diseases and multimorbidity. (unimputed data).

**Table S8.** Comparative performance metrics of seven machine learning algorithms (unimputed data).

**Table S9.** Cox proportional hazards regression analysis of the association between joint sleep–depression trajectories and the risk of incident chronic diseases and multimorbidity during the 2018–2020 follow-up period.

**Table S10.** Cox proportional hazards regression analysis comparing the risk of incident chronic diseases and multimorbidity between Group 2 and Group 3.

**Figure S1.** Flow diagram for participants included in the study.

**Figure S2.** Forest plot of joint sleep–depression trajectories and risk of multiple incident chronic diseases and multimorbidity (unimputed data).

**Figure S3.** Kaplan–Meier cumulative incidence curves for chronic diseases and multimorbidity stratified by joint trajectory groups(unimputed data).

**Figure S4.** Feature selection results based on unimputed data.

**Figure S5.** Comparative performance of seven predictive models using unimputed data.

**Figure S6.** SHAP-based interpretability for the top-performing model (unimputed data).

**Figure S7.** Forest plot of joint sleep–depression trajectories and risk of multiple incident chronic diseases and multimorbidity during the 2018–2020 follow-up period.

**Figure S8.** Web-based risk calculator for predicting membership in the high-risk joint sleep–depression trajectory.

**Table S1.** Definitions and measurement criteria for key study variables.

| **Variables** | **Description in CHARLS** |
| --- | --- |
| Social participation | Defined as participation in any social activities within the past month. |
| Social isolation score | Classified into four levels based on social environment: Low (strong support, frequent contact); Mild (slight limitations); Moderate (notable risk, limited engagement); and High (critical lack of support, minimal interaction). |
| Balance | Assessed by the ability to maintain a tandem stance (heel-to-toe) for up to 60 seconds. |
| Chair stand test | Based on the time required to complete five chair stands. Performance categorized via SPPB criteria: Low (≥13.7 s), Moderate (11.2–13.6 s), and High (≤11.1 s). |
| Lung function | Assessed via Peak Expiratory Flow (PEF). Categorized as: Low (≤400 L/min), Moderate (401–600 L/min), and High (≥601 L/min). |
| ADL | Yes: Ability to perform all basic self-care tasks (e.g.bathing, dressing, eating) without assistance.  No: Requiring assistance with at least one task. |
| IADL | Yes: Ability to perform all complex daily tasks (e.g., shopping, cooking, managing finances) without assistance.  No: Requiring assistance with at least one task. |
| Body pain | Defined by self-reported frequent pain and quantified by the number of painful sites (range: 0–15). Higher counts indicate greater pain burden. |
| Cognition score | A composite score (range: 0–21) summing episodic memory (0–10; immediate/delayed recall) and mental status (0–11; orientation, calculation, drawing). Higher scores indicate better cognitive function. |

**Abbreviations: ADL,** Activities of Daily Living; **IADL**, Instrumental Activities of Daily Living; **PEF**, Peak Expiratory Flow; **CHARLS**, China Health and Retirement Longitudinal Study.

**Table S2.** Goodness-of-fit statistics for joint trajectory models with 1 to 6 latent classes.

| **Number of classes** | **AIC** | **BIC** | **Group membership (%)** | | | | | | **Ave PP (%)** | | | | | |
| --- | --- | --- | --- | --- | --- | --- | --- | --- | --- | --- | --- | --- | --- | --- |
| 1 | 394678.5 | 394746.7 | 100 |  |  |  |  |  | 100 |  |  |  |  |  |
| 2 | 379483.7 | 379637.3 | 51.3 | 48.7 |  |  |  |  | 93.6 | 95.0 |  |  |  |  |
| 3 | 376407.2 | 376637.5 | 27.2 | 46.8 | 26.0 |  |  |  | 88.9 | 86.7 | 90.6 |  |  |  |
| **4** | **373540.3** | **373855.7** | **24.4** | **27.2** | **25.0** | **23.4** |  |  | **81.3** | **88.4** | **83.0** | **88.9** |  |  |
| 5 | 372755.3 | 372256.1 | 20.8 | 24.5 | 20.1 | 3.8 | 30.8 |  | 79.1 | 84.2 | 81.1 | 86.2 | 83.1 |  |
| 6 | 373640.5 | 374138.4 | 18.5 | 22.1 | 19.3 | 3.2 | 28.4 | 8.5 | 76.5 | 81.0 | 78.4 | 82.1 | 80.5 | 75.2 |

**Abbreviations:AIC**, Akaike Information Criterion; **BIC**, Bayesian Information Criterion;

**Ave PP**, Average Posterior Probability.

**Table S3.** Baseline characteristics of participants.

| **Variables** | **Total** (n = 3221) | **G1** (n = 788) | **G2** (n = 875) | **G3** (n = 805) | **G4** (n = 753) | *P* |
| --- | --- | --- | --- | --- | --- | --- |
| **Demographic Characteristics** |  |  |  |  |  |  |
| Age, Mean ± SD | 65.80 ± 4.93 | 65.59 ± 4.78 | 66.08 ± 5.17 | 65.62 ± 4.93 | 65.88 ± 4.80 | 0.130 |
| Gender, n(%) |  |  |  |  |  | <.001 |
| Female | 1596 (49.55) | 288 (36.55) | 409 (46.74) | 398 (49.44) | 501 (66.53) |  |
| Male | 1625 (50.45) | 500 (63.45) | 466 (53.26) | 407 (50.56) | 252 (33.47) |  |
| Education, n(%) |  |  |  |  |  | <.001 |
| Primary School | 3022 (93.82) | 694 (88.07) | 814 (93.03) | 780 (96.89) | 734 (97.48) |  |
| Secondary School | 154 (4.78) | 66 (8.38) | 48 (5.49) | 24 (2.98) | 16 (2.12) |  |
| College and Above | 45 (1.40) | 28 (3.55) | 13 (1.49) | 1 (0.12) | 3 (0.40) |  |
| Marital status, n(%) |  |  |  |  |  | <.001 |
| Have spouse | 2718 (84.38) | 706 (89.59) | 737 (84.23) | 668 (82.98) | 607 (80.61) |  |
| No spouse | 503 (15.62) | 82 (10.41) | 138 (15.77) | 137 (17.02) | 146 (19.39) |  |
| Hukou, n(%) |  |  |  |  |  | <.001 |
| Agriculture | 2577 (80.01) | 546 (69.29) | 658 (75.20) | 695 (86.34) | 678 (90.04) |  |
| Non-agriculture | 617 (19.16) | 233 (29.57) | 207 (23.66) | 104 (12.92) | 73 (9.69) |  |
| Unified residence | 27 (0.84) | 9 (1.14) | 10 (1.14) | 6 (0.75) | 2 (0.27) |  |
| Residence, n(%) |  |  |  |  |  | <.001 |
| Urban | 1060 (32.91) | 350 (44.42) | 342 (39.09) | 206 (25.59) | 162 (21.51) |  |
| Rural | 2161 (67.09) | 438 (55.58) | 533 (60.91) | 599 (74.41) | 591 (78.49) |  |
| Region, n(%) |  |  |  |  |  | <.001 |
| Western | 1071 (33.25) | 181 (22.97) | 265 (30.29) | 274 (34.04) | 351 (46.61) |  |
| Eastern | 1445 (44.86) | 420 (53.30) | 410 (46.86) | 364 (45.22) | 251 (33.33) |  |
| Central | 514 (15.96) | 130 (16.50) | 153 (17.49) | 119 (14.78) | 112 (14.87) |  |
| Northeast | 191 (5.93) | 57 (7.23) | 47 (5.37) | 48 (5.96) | 39 (5.18) |  |
| **Socioeconomic Factors** |  |  |  |  |  |  |
| Retirement, n(%) |  |  |  |  |  | <.001 |
| No | 2669 (82.86) | 560 (71.07) | 698 (79.77) | 721 (89.57) | 690 (91.63) |  |
| Yes | 552 (17.14) | 228 (28.93) | 177 (20.23) | 84 (10.43) | 63 (8.37) |  |
| Medical insurance, n(%) |  |  |  |  |  | 0.039 |
| No | 150 (4.66) | 24 (3.05) | 40 (4.57) | 40 (4.97) | 46 (6.11) |  |
| Yes | 3071 (95.34) | 764 (96.95) | 835 (95.43) | 765 (95.03) | 707 (93.89) |  |
| HPCC, Mean ± SD | 6216.07 ± 7608.53 | 7314.83 ± 9941.84 | 6690.13 ± 8232.85 | 5490.73 ± 5993.31 | 5290.81 ± 4930.70 | <.001 |
| Life satisfaction, n(%) |  |  |  |  |  | <.001 |
| Not satisfied | 77 (2.39) | 2 (0.25) | 5 (0.57) | 18 (2.24) | 52 (6.91) |  |
| Slightly dissatisfied | 386 (11.98) | 31 (3.93) | 72 (8.23) | 121 (15.03) | 162 (21.51) |  |
| Moderately satisfied | 2006 (62.28) | 508 (64.47) | 579 (66.17) | 486 (60.37) | 433 (57.50) |  |
| Very satisfied | 694 (21.55) | 227 (28.81) | 205 (23.43) | 165 (20.50) | 97 (12.88) |  |
| Extremely satisfied | 58 (1.80) | 20 (2.54) | 14 (1.60) | 15 (1.86) | 9 (1.20) |  |
| **Lifestyle and Behaviors** |  |  |  |  |  |  |
| Smoking Status, n(%) |  |  |  |  |  | <.001 |
| Never smoked | 1865 (57.90) | 425 (53.93) | 498 (56.91) | 450 (55.90) | 492 (65.34) |  |
| Former smoker | 331 (10.28) | 89 (11.29) | 92 (10.51) | 75 (9.32) | 75 (9.96) |  |
| Current smoker | 1025 (31.82) | 274 (34.77) | 285 (32.57) | 280 (34.78) | 186 (24.70) |  |
| Alcohol Consumption, n(%) |  |  |  |  |  | <.001 |
| Non-drinker | 1887 (58.58) | 436 (55.33) | 484 (55.31) | 487 (60.50) | 480 (63.75) |  |
| Occasional drinker | 356 (11.05) | 74 (9.39) | 90 (10.29) | 100 (12.42) | 92 (12.22) |  |
| Frequent drinker | 978 (30.36) | 278 (35.28) | 301 (34.40) | 218 (27.08) | 181 (24.04) |  |
| Nap duration, Mean ± SD | 33.34 ± 43.07 | 39.84 ± 45.92 | 30.47 ± 40.85 | 37.83 ± 45.34 | 25.07 ± 38.15 | <.001 |
| Social participation, n(%) |  |  |  |  |  | <.001 |
| No | 1741 (54.05) | 364 (46.19) | 454 (51.89) | 456 (56.65) | 467 (62.02) |  |
| Yes | 1480 (45.95) | 424 (53.81) | 421 (48.11) | 349 (43.35) | 286 (37.98) |  |
| Social isolation score, n(%) |  |  |  |  |  | <.001 |
| Low | 1120 (34.77) | 346 (43.91) | 325 (37.14) | 266 (33.04) | 183 (24.30) |  |
| Mild | 1573 (48.84) | 355 (45.05) | 394 (45.03) | 402 (49.94) | 422 (56.04) |  |
| Moderate | 395 (12.26) | 65 (8.25) | 111 (12.69) | 105 (13.04) | 114 (15.14) |  |
| High | 133 (4.13) | 22 (2.79) | 45 (5.14) | 32 (3.98) | 34 (4.52) |  |
| **Health Status Indicators** |  |  |  |  |  |  |
| BMI, Mean ± SD | 23.10 ± 3.69 | 23.68 ± 3.61 | 23.10 ± 3.61 | 23.06 ± 3.77 | 22.52 ± 3.70 | <.001 |
| Waist circumference, Mean ± SD | 84.36 ± 12.31 | 85.93 ± 11.86 | 84.59 ± 12.92 | 84.40 ± 12.16 | 82.40 ± 11.97 | <.001 |
| SBP, Mean ± SD | 131.50 ± 21.12 | 132.11 ± 20.38 | 131.85 ± 21.61 | 131.16 ± 20.98 | 130.79 ± 21.45 | 0.583 |
| DBP, Mean ± SD | 74.42 ± 11.47 | 75.11 ± 11.43 | 74.39 ± 11.54 | 74.25 ± 11.53 | 73.90 ± 11.35 | 0.204 |
| Grip strength,  Mean ± SD | 30.30 ± 10.15 | 33.81 ± 9.59 | 30.48 ± 9.91 | 29.68 ± 9.99 | 27.08 ± 9.99 | <.001 |
| Balance, n(%) |  |  |  |  |  | <.001 |
| No | 793 (24.62) | 144 (18.27) | 198 (22.63) | 218 (27.08) | 233 (30.94) |  |
| Yes | 2428 (75.38) | 644 (81.73) | 677 (77.37) | 587 (72.92) | 520 (69.06) |  |
| Chair stand test, n(%) |  |  |  |  |  | <.001 |
| Low | 583 (18.10) | 91 (11.55) | 146 (16.69) | 152 (18.88) | 194 (25.76) |  |
| Moderate | 689 (21.39) | 136 (17.26) | 204 (23.31) | 170 (21.12) | 179 (23.77) |  |
| High | 1949 (60.51) | 561 (71.19) | 525 (60.00) | 483 (60.00) | 380 (50.46) |  |
| Lung function, n(%) |  |  |  |  |  | <.001 |
| Low | 2749 (85.35) | 620 (78.68) | 733 (83.77) | 702 (87.20) | 694 (92.16) |  |
| Moderate | 441 (13.69) | 156 (19.80) | 135 (15.43) | 94 (11.68) | 56 (7.44) |  |
| High | 31 (0.96) | 12 (1.52) | 7 (0.80) | 9 (1.12) | 3 (0.40) |  |
| ADL, n(%) |  |  |  |  |  | <.001 |
| No | 2595 (80.57) | 737 (93.53) | 744 (85.03) | 661 (82.11) | 453 (60.16) |  |
| Yes | 626 (19.43) | 51 (6.47) | 131 (14.97) | 144 (17.89) | 300 (39.84) |  |
| IADL, n(%) |  |  |  |  |  | <.001 |
| No | 2492 (77.37) | 706 (89.59) | 730 (83.43) | 598 (74.29) | 458 (60.82) |  |
| Yes | 729 (22.63) | 82 (10.41) | 145 (16.57) | 207 (25.71) | 295 (39.18) |  |
| Disability, n(%) |  |  |  |  |  | <.001 |
| No | 2614 (81.15) | 682 (86.55) | 730 (83.43) | 646 (80.25) | 556 (73.84) |  |
| Yes | 607 (18.85) | 106 (13.45) | 145 (16.57) | 159 (19.75) | 197 (26.16) |  |
| Vision, n(%) |  |  |  |  |  | <.001 |
| Poor | 895 (27.79) | 118 (14.97) | 205 (23.43) | 241 (29.94) | 331 (43.96) |  |
| Fair | 1289 (40.02) | 313 (39.72) | 382 (43.66) | 334 (41.49) | 260 (34.53) |  |
| Good | 709 (22.01) | 232 (29.44) | 201 (22.97) | 164 (20.37) | 112 (14.87) |  |
| Very Good | 291 (9.03) | 113 (14.34) | 76 (8.69) | 57 (7.08) | 45 (5.98) |  |
| Excellent | 37 (1.15) | 12 (1.52) | 11 (1.26) | 9 (1.12) | 5 (0.66) |  |
| Hearing, n(%) |  |  |  |  |  | <.001 |
| Poor | 515 (15.99) | 78 (9.90) | 131 (14.97) | 142 (17.64) | 164 (21.78) |  |
| Fair | 1451 (45.05) | 305 (38.71) | 413 (47.20) | 353 (43.85) | 380 (50.46) |  |
| Good | 878 (27.26) | 265 (33.63) | 240 (27.43) | 220 (27.33) | 153 (20.32) |  |
| Very Good | 341 (10.59) | 123 (15.61) | 85 (9.71) | 81 (10.06) | 52 (6.91) |  |
| Excellent | 36 (1.12) | 17 (2.16) | 6 (0.69) | 9 (1.12) | 4 (0.53) |  |
| Teeth loss, n(%) |  |  |  |  |  | 0.048 |
| No | 2830 (87.86) | 708 (89.85) | 773 (88.34) | 707 (87.83) | 642 (85.26) |  |
| Yes | 391 (12.14) | 80 (10.15) | 102 (11.66) | 98 (12.17) | 111 (14.74) |  |
| SRH, n(%) |  |  |  |  |  | <.001 |
| Poor | 134 (4.16) | 6 (0.76) | 21 (2.40) | 34 (4.22) | 73 (9.69) |  |
| Fair | 796 (24.71) | 63 (7.99) | 192 (21.94) | 230 (28.57) | 311 (41.30) |  |
| Average | 1623 (50.39) | 422 (53.55) | 489 (55.89) | 401 (49.81) | 311 (41.30) |  |
| Good | 495 (15.37) | 213 (27.03) | 130 (14.86) | 107 (13.29) | 45 (5.98) |  |
| Very Good | 173 (5.37) | 84 (10.66) | 43 (4.91) | 33 (4.10) | 13 (1.73) |  |
| Body pain, Mean ± SD | 1.51 ± 2.88 | 0.31 ± 1.18 | 1.05 ± 2.27 | 1.50 ± 2.65 | 3.30 ± 3.94 | <.001 |
| History of falls, n(%) |  |  |  |  |  | <.001 |
| No | 2624 (81.47) | 705 (89.47) | 718 (82.06) | 675 (83.85) | 526 (69.85) |  |
| Yes | 597 (18.53) | 83 (10.53) | 157 (17.94) | 130 (16.15) | 227 (30.15) |  |
| Cognition score, Mean ± SD | 10.36 ± 4.35 | 12.15 ± 3.92 | 10.89 ± 4.03 | 9.77 ± 4.29 | 8.49 ± 4.35 | <.001 |
| Chronic diseases count, Mean ± SD | 1.54 ± 1.41 | 1.12 ± 1.19 | 1.56 ± 1.38 | 1.47 ± 1.32 | 2.03 ± 1.58 | <.001 |

**Note:** Data are presented as mean ± standard deviation (SD) for continuous variables and frequency (percentage) for categorical variables. P values were derived from one-way ANOVA for continuous variables and Chi-square tests for categorical variables.

**Abbreviations:** **BMI**, Body Mass Index; **SBP**, Systolic Blood Pressure; **DBP**, Diastolic Blood Pressure; **HPCC**, Household Per Capita Consumption expenditure; **ADL**, Activities of Daily Living; **IADL**, Instrumental Activities of Daily Living; **SRH**, Self-Rated Health;

**Group definitions: G1**, normal-stable sleep and low-stable depression; **G2**, short-stable sleep and low-stable depression; **G3**, normal-increasing sleep and moderate-increasing depression; **G4**, short-decreasing sleep and high-increasing depression.

**Table S4.** Cox proportional hazards regression analysis of the association between joint sleep–depression trajectories and the risk of incident chronic diseases and multimorbidity.

| **Disease / Group** | **Model 1** | | **Model 2** | | **Model 3** | |
| --- | --- | --- | --- | --- | --- | --- |
|  | HR (95% CI) | *P* | HR (95% CI) | *P* | HR (95% CI) | *P* |
| **Hypertension** |  |  |  |  |  |  |
| G1 | Ref | - | Ref | - | Ref | - |
| G2 | 1.08 (0.95, 1.22) | 0.253 | 1.08 (0.95, 1.22) | 0.254 | 1.12 (0.99, 1.27) | 0.085 |
| G3 | 1.19 (1.05, 1.35) | 0.006 | 1.20 (1.06, 1.36) | 0.004 | 1.25 (1.09, 1.42) | 0.001 |
| G4 | 1.44 (1.26, 1.64) | <0.001 | 1.45 (1.26, 1.65) | <0.001 | 1.48 (1.27, 1.73) | <0.001 |
| **Dyslipidemia** |  |  |  |  |  |  |
| G1 | Ref | - | Ref | - | Ref | - |
| G2 | 1.22 (1.08, 1.39) | 0.002 | 1.25 (1.10, 1.42) | 0.001 | 1.22 (1.07, 1.40) | 0.003 |
| G3 | 1.36 (1.19, 1.55) | <0.001 | 1.37 (1.20, 1.56) | <0.001 | 1.34 (1.17, 1.53) | <0.001 |
| G4 | 1.80 (1.57, 2.07) | <0.001 | 1.86 (1.62, 2.13) | <0.001 | 1.78 (1.53, 2.08) | <0.001 |
| **Diabetes** |  |  |  |  |  |  |
| G1 | Ref | - | Ref | - | Ref | - |
| G2 | 1.29 (1.08, 1.53) | 0.004 | 1.32 (1.11, 1.57) | 0.002 | 1.27 (1.06, 1.51) | 0.008 |
| G3 | 1.25 (1.04, 1.49) | 0.015 | 1.26 (1.06, 1.51) | 0.01 | 1.17 (0.97, 1.41) | 0.103 |
| G4 | 1.85 (1.55, 2.21) | <0.001 | 1.93 (1.61, 2.31) | <0.001 | 1.63 (1.32, 1.99) | <0.001 |
| **Heart disease** |  |  |  |  |  |  |
| G1 | Ref | - | Ref | - | Ref | - |
| G2 | 1.50 (1.28, 1.75) | <0.001 | 1.52 (1.30, 1.78) | <0.001 | 1.41 (1.21, 1.66) | <0.001 |
| G3 | 1.51 (1.29, 1.77) | <0.001 | 1.52 (1.30, 1.78) | <0.001 | 1.37 (1.16, 1.61) | <0.001 |
| G4 | 2.18 (1.85, 2.56) | <0.001 | 2.24 (1.90, 2.64) | <0.001 | 1.82 (1.52, 2.19) | <0.001 |
| **Stroke** |  |  |  |  |  |  |
| G1 | Ref | - | Ref | - | Ref | - |
| G2 | 1.38 (1.09, 1.75) | 0.007 | 1.39 (1.10, 1.76) | 0.006 | 1.31 (1.03, 1.67) | 0.027 |
| G3 | 1.83 (1.45, 2.30) | <0.001 | 1.83 (1.45, 2.30) | <0.001 | 1.68 (1.32, 2.13) | <0.001 |
| G4 | 3.04 (2.42, 3.83) | <0.001 | 3.08 (2.44, 3.87) | <0.001 | 2.56 (1.98, 3.30) | <0.001 |
| **Chronic lung disease** |  |  |  |  |  |  |
| G1 | Ref | - | Ref | - | Ref | - |
| G2 | 1.70 (1.42, 2.03) | <0.001 | 1.69 (1.41, 2.03) | <0.001 | 1.51 (1.25, 1.81) | <0.001 |
| G3 | 1.99 (1.67, 2.38) | <0.001 | 1.98 (1.65, 2.37) | <0.001 | 1.71 (1.42, 2.06) | <0.001 |
| G4 | 3.31 (2.76, 3.96) | <0.001 | 3.27 (2.73, 3.93) | <0.001 | 2.35 (1.92, 2.88) | <0.001 |
| **Asthma** |  |  |  |  |  |  |
| G1 | Ref | - | Ref | - | Ref | - |
| G2 | 1.50 (1.11, 2.03) | 0.009 | 1.49 (1.10, 2.01) | 0.011 | 1.32 (0.97, 1.80) | 0.077 |
| G3 | 1.63 (1.20, 2.21) | 0.002 | 1.59 (1.17, 2.15) | 0.003 | 1.39 (1.01, 1.90) | 0.041 |
| G4 | 2.91 (2.16, 3.91) | <0.001 | 2.83 (2.10, 3.81) | <0.001 | 2.23 (1.61, 3.10) | <0.001 |
| **Liver disease** |  |  |  |  |  |  |
| G1 | Ref | - | Ref | - | Ref | - |
| G2 | 1.18 (0.94, 1.47) | 0.158 | 1.19 (0.95, 1.49) | 0.129 | 1.05 (0.83, 1.32) | 0.691 |
| G3 | 1.31 (1.05, 1.65) | 0.017 | 1.33 (1.06, 1.67) | 0.013 | 1.12 (0.89, 1.42) | 0.338 |
| G4 | 2.10 (1.67, 2.63) | <0.001 | 2.15 (1.71, 2.70) | <0.001 | 1.50 (1.16, 1.95) | 0.002 |
| **Cancer** |  |  |  |  |  |  |
| G1 | Ref | - | Ref | - | Ref | - |
| G2 | 1.54 (1.05, 2.26) | 0.029 | 1.48 (1.01, 2.18) | 0.047 | 1.37 (0.92, 2.03) | 0.122 |
| G3 | 1.43 (0.95, 2.14) | 0.084 | 1.41 (0.94, 2.12) | 0.095 | 1.26 (0.82, 1.92) | 0.292 |
| G4 | 2.03 (1.35, 3.05) | 0.001 | 1.90 (1.26, 2.86) | 0.002 | 1.56 (1.05, 2.31) | 0.029 |
| **Digestive disease** |  |  |  |  |  |  |
| G1 | Ref | - | Ref | - | Ref | - |
| G2 | 1.40 (1.21, 1.62) | <0.001 | 1.40 (1.21, 1.62) | <0.001 | 1.23 (1.06, 1.43) | 0.006 |
| G3 | 1.46 (1.26, 1.69) | <0.001 | 1.47 (1.27, 1.70) | <0.001 | 1.23 (1.06, 1.43) | 0.008 |
| G4 | 2.31 (1.98, 2.69) | <0.001 | 2.31 (1.99, 2.70) | <0.001 | 1.66 (1.40, 1.97) | <0.001 |
| **Kidney disease** |  |  |  |  |  |  |
| G1 | Ref | - | Ref | - | Ref | - |
| G2 | 1.84 (1.51, 2.25) | <0.001 | 1.84 (1.51, 2.25) | <0.001 | 1.56 (1.27, 1.91) | <0.001 |
| G3 | 1.81 (1.47, 2.23) | <0.001 | 1.84 (1.49, 2.26) | <0.001 | 1.42 (1.15, 1.77) | 0.001 |
| G4 | 3.28 (2.67, 4.03) | <0.001 | 3.30 (2.68, 4.06) | <0.001 | 2.00 (1.58, 2.52) | <0.001 |
| **Arthritis** |  |  |  |  |  |  |
| G1 | Ref | - | Ref | - | Ref | - |
| G2 | 1.50 (1.31, 1.70) | <0.001 | 1.49 (1.31, 1.70) | <0.001 | 1.35 (1.18, 1.54) | <0.001 |
| G3 | 1.52 (1.33, 1.74) | <0.001 | 1.52 (1.33, 1.74) | <0.001 | 1.29 (1.13, 1.49) | <0.001 |
| G4 | 2.07 (1.79, 2.40) | <0.001 | 2.05 (1.77, 2.38) | <0.001 | 1.50 (1.27, 1.77) | <0.001 |
| **Memory-related disorders** |  |  |  |  |  |  |
| G1 | Ref | - | Ref | - | Ref | - |
| G2 | 1.93 (1.44, 2.60) | <0.001 | 1.92 (1.43, 2.58) | <0.001 | 1.63 (1.21, 2.21) | 0.001 |
| G3 | 2.44 (1.81, 3.27) | <0.001 | 2.48 (1.84, 3.33) | <0.001 | 1.97 (1.46, 2.68) | <0.001 |
| G4 | 4.77 (3.58, 6.34) | <0.001 | 4.76 (3.57, 6.35) | <0.001 | 3.08 (2.25, 4.22) | <0.001 |
| **Multimorbidity** |  |  |  |  |  |  |
| G1 | Ref | - | Ref | - | Ref | - |
| G2 | 1.40 (1.27, 1.54) | <0.001 | 1.40 (1.27, 1.55) | <0.001 | 1.31 (1.19, 1.45) | <0.001 |
| G3 | 1.50 (1.36, 1.66) | <0.001 | 1.51 (1.37, 1.67) | <0.001 | 1.37 (1.24, 1.52) | <0.001 |
| G4 | 2.35 (2.10, 2.63) | <0.001 | 2.37 (2.12, 2.66) | <0.001 | 1.97 (1.74, 2.23) | <0.001 |

**Note:** Data are presented as Hazard Ratio (95% Confidence Interval).

Model 1: Adjusted for age, sex, educational attainment, marital status, Hukou status, residence, and geographic region.

Model 2: Additionally adjusted for smoking status, alcohol consumption, nap duration, social participation, and social isolation.

Model 3: Further adjusted for body mass index (BMI), waist circumference, blood pressure, grip strength, balance, chair stand test, lung function, ADL/IADL disability, sensory function, tooth loss, self-rated health, body pain, history of falls, cognitive function, and baseline chronic disease count.

**Group definitions: G1**, normal-stable sleep and low-stable depression; **G2**, short-stable sleep and low-stable depression; **G3**, normal-increasing sleep and moderate-increasing depression; **G4**, short-decreasing sleep and high-increasing depression.

**Abbreviations: HR**, Hazard Ratio; **CI**, Confidence Interval; **Ref**, Reference group.

**Table S5.** Comparative performance metrics of seven machine learning algorithms in the validation set.

| **Model** | **AUC** | **Accuracy** | **Precision** | **Sensitivity** | **Specificity** | **F1 Score** |
| --- | --- | --- | --- | --- | --- | --- |
| Logistic | 0.8035 | 0.8193 | 0.6413 | 0.2911 | 0.9647 | 0.4123 |
| Decision Tree | 0.7581 | 0.8071 | 0.5556 | 0.2669 | 0.9475 | 0.4014 |
| Random Forest | 0.7816 | 0.8123 | 0.5874 | 0.2789 | 0.9536 | 0.4094 |
| XGBoost | 0.8054 | 0.8201 | 0.6514 | 0.3010 | 0.9652 | 0.4228 |
| LightGBM | 0.7889 | 0.8115 | 0.5749 | 0.2806 | 0.9470 | 0.4232 |
| SVM | 0.7993 | 0.8127 | 0.602 | 0.2732 | 0.9606 | 0.3862 |
| ANN | 0.8051 | 0.8211 | 0.6473 | 0.2972 | 0.9632 | 0.4279 |

**Abbreviations: AUC**, Area Under the Receiver Operating Characteristic Curve; **SVM**, Support Vector Machine; **ANN**, Artificial Neural Network; **XGBoost**, eXtreme Gradient Boosting; **LightGBM**, Light Gradient Boosting Machine.

**Table S6.** Baseline characteristics of participants (unimputed data).

| **Variables** | Total (n = 1989) | G1 (n = 510) | G2 (n = 546) | G3 (n = 502) | G4 (n = 431) | P |
| --- | --- | --- | --- | --- | --- | --- |
| **Demographic Characteristics** |  |  |  |  |  |  |
| Age, Mean ± SD | 65.46 ± 4.67 | 65.17 ± 4.57 | 65.62 ± 4.80 | 65.51 ± 4.84 | 65.55 ± 4.43 | 0.428 |
| Gender, n(%) |  |  |  |  |  | <.001 |
| Female | 924 (46.46) | 180 (35.29) | 241 (44.14) | 235 (46.81) | 268 (62.18) |  |
| Male | 1065 (53.54) | 330 (64.71) | 305 (55.86) | 267 (53.19) | 163 (37.82) |  |
| Education, n(%) |  |  |  |  |  | <.001 |
| Primary School | 1872 (94.12) | 452 (88.63) | 507 (92.86) | 489 (97.41) | 424 (98.38) |  |
| Secondary School | 93 (4.68) | 44 (8.63) | 30 (5.49) | 12 (2.39) | 7 (1.62) |  |
| College and Above | 24 (1.21) | 14 (2.75) | 9 (1.65) | 1 (0.20) | 0 (0.00) |  |
| Marital status, n(%) |  |  |  |  |  | 0.005 |
| Have spouse | 1719 (86.43) | 462 (90.59) | 474 (86.81) | 424 (84.46) | 359 (83.29) |  |
| No spouse | 270 (13.57) | 48 (9.41) | 72 (13.19) | 78 (15.54) | 72 (16.71) |  |
| Hukou, n(%) |  |  |  |  |  | <.001 |
| Agriculture | 1597 (80.29) | 351 (68.82) | 422 (77.29) | 433 (86.25) | 391 (90.72) |  |
| Non-agriculture | 377 (18.95) | 153 (30.00) | 119 (21.79) | 65 (12.95) | 40 (9.28) |  |
| Unified residence | 15 (0.75) | 6 (1.18) | 5 (0.92) | 4 (0.80) | 0 (0.00) |  |
| Residence, n(%) |  |  |  |  |  | <.001 |
| Urban | 612 (30.77) | 219 (42.94) | 194 (35.53) | 114 (22.71) | 85 (19.72) |  |
| Rural | 1377 (69.23) | 291 (57.06) | 352 (64.47) | 388 (77.29) | 346 (80.28) |  |
| Region, n(%) |  |  |  |  |  | <.001 |
| Western | 644 (32.38) | 115 (22.55) | 161 (29.49) | 166 (33.07) | 202 (46.87) |  |
| Eastern | 850 (42.74) | 265 (51.96) | 246 (45.05) | 208 (41.43) | 131 (30.39) |  |
| Central | 352 (17.70) | 84 (16.47) | 104 (19.05) | 87 (17.33) | 77 (17.87) |  |
| Northeast | 143 (7.19) | 46 (9.02) | 35 (6.41) | 41 (8.17) | 21 (4.87) |  |
| **Socioeconomic Factors** |  |  |  |  |  |  |
| Retirement, n(%) |  |  |  |  |  | <.001 |
| No | 1654 (83.16) | 358 (70.20) | 449 (82.23) | 450 (89.64) | 397 (92.11) |  |
| Yes | 335 (16.84) | 152 (29.80) | 97 (17.77) | 52 (10.36) | 34 (7.89) |  |
| Medical insurance, n(%) |  |  |  |  |  | 0.135 |
| No | 92 (4.63) | 15 (2.94) | 25 (4.58) | 26 (5.18) | 26 (6.03) |  |
| Yes | 1897 (95.37) | 495 (97.06) | 521 (95.42) | 476 (94.82) | 405 (93.97) |  |
| HPCC, Mean ± SD | 6073.77 ± 6693.55 | 6761.52 ± 6337.77 | 6737.56 ± 8430.05 | 5528.45 ± 6204.80 | 5054.21 ± 4706.83 | <.001 |
| Life satisfaction, n(%) |  |  |  |  |  | <.001 |
| Not satisfied | 41 (2.06) | 1 (0.20) | 2 (0.37) | 9 (1.79) | 29 (6.73) |  |
| Slightly dissatisfied | 212 (10.66) | 16 (3.14) | 38 (6.96) | 68 (13.55) | 90 (20.88) |  |
| Moderately satisfied | 1239 (62.29) | 328 (64.31) | 359 (65.75) | 307 (61.16) | 245 (56.84) |  |
| Very satisfied | 456 (22.93) | 149 (29.22) | 138 (25.27) | 107 (21.31) | 62 (14.39) |  |
| Extremely satisfied | 41 (2.06) | 16 (3.14) | 9 (1.65) | 11 (2.19) | 5 (1.16) |  |
| **Lifestyle and Behaviors** |  |  |  |  |  |  |
| Smoking Status, n(%) |  |  |  |  |  | 0.054 |
| Never smoked | 1117 (56.16) | 275 (53.92) | 307 (56.23) | 266 (52.99) | 269 (62.41) |  |
| Former smoker | 215 (10.81) | 56 (10.98) | 57 (10.44) | 54 (10.76) | 48 (11.14) |  |
| Current smoker | 657 (33.03) | 179 (35.10) | 182 (33.33) | 182 (36.25) | 114 (26.45) |  |
| Alcohol Consumption, n(%) |  |  |  |  |  | <.001 |
| Non-drinker | 1116 (56.11) | 269 (52.75) | 292 (53.48) | 294 (58.57) | 261 (60.56) |  |
| Occasional drinker | 241 (12.12) | 50 (9.80) | 57 (10.44) | 70 (13.94) | 64 (14.85) |  |
| Frequent drinker | 632 (31.77) | 191 (37.45) | 197 (36.08) | 138 (27.49) | 106 (24.59) |  |
| Nap duration, Mean ± SD | 33.58 ± 43.20 | 39.75 ± 45.42 | 30.02 ± 41.61 | 37.36 ± 44.59 | 26.38 ± 39.34 | <.001 |
| Social participation, n(%) |  |  |  |  |  | <.001 |
| No | 1023 (51.43) | 226 (44.31) | 265 (48.53) | 269 (53.59) | 263 (61.02) |  |
| Yes | 966 (48.57) | 284 (55.69) | 281 (51.47) | 233 (46.41) | 168 (38.98) |  |
| Social isolation score, n(%) |  |  |  |  |  | <.001 |
| Low | 747 (37.56) | 233 (45.69) | 224 (41.03) | 182 (36.25) | 108 (25.06) |  |
| Mild | 958 (48.16) | 223 (43.73) | 239 (43.77) | 243 (48.41) | 253 (58.70) |  |
| Moderate | 213 (10.71) | 44 (8.63) | 58 (10.62) | 56 (11.16) | 55 (12.76) |  |
| High | 71 (3.57) | 10 (1.96) | 25 (4.58) | 21 (4.18) | 15 (3.48) |  |
| **Health Status Indicators** |  |  |  |  |  |  |
| BMI, Mean ± SD | 23.05 ± 3.56 | 23.60 ± 3.37 | 23.08 ± 3.48 | 23.00 ± 3.62 | 22.42 ± 3.71 | <.001 |
| Waist circumference, Mean ± SD | 84.56 ± 11.67 | 86.40 ± 10.88 | 85.01 ± 11.78 | 84.36 ± 11.65 | 82.03 ± 12.02 | <.001 |
| SBP, Mean ± SD | 131.18 ± 20.97 | 132.49 ± 19.94 | 131.43 ± 21.98 | 130.57 ± 20.38 | 130.02 ± 21.51 | 0.283 |
| DBP, Mean ± SD | 73.88 ± 11.27 | 74.91 ± 11.24 | 73.85 ± 11.31 | 73.39 ± 11.28 | 73.27 ± 11.20 | 0.091 |
| Grip strength,  Mean ± SD | 31.17 ± 9.86 | 34.09 ± 9.42 | 31.53 ± 9.82 | 30.49 ± 9.84 | 28.07 ± 9.43 | <.001 |
| Balance, n(%) |  |  |  |  |  | 0.008 |
| No | 466 (23.43) | 100 (19.61) | 117 (21.43) | 128 (25.50) | 121 (28.07) |  |
| Yes | 1523 (76.57) | 410 (80.39) | 429 (78.57) | 374 (74.50) | 310 (71.93) |  |
| Chair stand test, n(%) |  |  |  |  |  | <.001 |
| Low | 335 (16.84) | 57 (11.18) | 84 (15.38) | 94 (18.73) | 100 (23.20) |  |
| Moderate | 434 (21.82) | 92 (18.04) | 121 (22.16) | 112 (22.31) | 109 (25.29) |  |
| High | 1220 (61.34) | 361 (70.78) | 341 (62.45) | 296 (58.96) | 222 (51.51) |  |
| Lung function, n(%) |  |  |  |  |  | <.001 |
| Low | 1686 (84.77) | 409 (80.20) | 447 (81.87) | 433 (86.25) | 397 (92.11) |  |
| Moderate | 289 (14.53) | 97 (19.02) | 96 (17.58) | 64 (12.75) | 32 (7.42) |  |
| High | 14 (0.70) | 4 (0.78) | 3 (0.55) | 5 (1.00) | 2 (0.46) |  |
| ADL, n(%) |  |  |  |  |  | <.001 |
| No | 1651 (83.01) | 479 (93.92) | 473 (86.63) | 426 (84.86) | 273 (63.34) |  |
| Yes | 338 (16.99) | 31 (6.08) | 73 (13.37) | 76 (15.14) | 158 (36.66) |  |
| IADL, n(%) |  |  |  |  |  | <.001 |
| No | 1589 (79.89) | 456 (89.41) | 465 (85.16) | 391 (77.89) | 277 (64.27) |  |
| Yes | 400 (20.11) | 54 (10.59) | 81 (14.84) | 111 (22.11) | 154 (35.73) |  |
| Disability, n(%) |  |  |  |  |  | <.001 |
| No | 1625 (81.70) | 437 (85.69) | 466 (85.35) | 401 (79.88) | 321 (74.48) |  |
| Yes | 364 (18.30) | 73 (14.31) | 80 (14.65) | 101 (20.12) | 110 (25.52) |  |
| Vision, n(%) |  |  |  |  |  | <.001 |
| Poor | 512 (25.74) | 78 (15.29) | 116 (21.25) | 143 (28.49) | 175 (40.60) |  |
| Fair | 821 (41.28) | 207 (40.59) | 251 (45.97) | 205 (40.84) | 158 (36.66) |  |
| Good | 427 (21.47) | 140 (27.45) | 120 (22.00) | 104 (20.72) | 63 (14.62) |  |
| Very Good | 202 (10.16) | 76 (14.90) | 52 (9.52) | 43 (8.57) | 31 (7.19) |  |
| Excellent | 27 (1.36) | 9 (1.76) | 7 (1.28) | 7 (1.39) | 4 (0.93) |  |
| Hearing, n(%) |  |  |  |  |  | <.001 |
| Poor | 287 (14.43) | 45 (8.82) | 76 (13.92) | 81 (16.14) | 85 (19.72) |  |
| Fair | 906 (45.55) | 199 (39.02) | 263 (48.17) | 224 (44.62) | 220 (51.04) |  |
| Good | 543 (27.30) | 169 (33.14) | 148 (27.11) | 136 (27.09) | 90 (20.88) |  |
| Very Good | 231 (11.61) | 86 (16.86) | 56 (10.26) | 55 (10.96) | 34 (7.89) |  |
| Excellent | 22 (1.11) | 11 (2.16) | 3 (0.55) | 6 (1.20) | 2 (0.46) |  |
| Teeth loss, n(%) |  |  |  |  |  | 0.143 |
| No | 1756 (88.29) | 460 (90.20) | 488 (89.38) | 438 (87.25) | 370 (85.85) |  |
| Yes | 233 (11.71) | 50 (9.80) | 58 (10.62) | 64 (12.75) | 61 (14.15) |  |
| SRH, n(%) |  |  |  |  |  | <.001 |
| Poor | 58 (2.92) | 2 (0.39) | 7 (1.28) | 17 (3.39) | 32 (7.42) |  |
| Fair | 463 (23.28) | 42 (8.24) | 116 (21.25) | 131 (26.10) | 174 (40.37) |  |
| Average | 1039 (52.24) | 281 (55.10) | 313 (57.33) | 255 (50.80) | 190 (44.08) |  |
| Good | 309 (15.54) | 128 (25.10) | 84 (15.38) | 74 (14.74) | 23 (5.34) |  |
| Very Good | 120 (6.03) | 57 (11.18) | 26 (4.76) | 25 (4.98) | 12 (2.78) |  |
| Body pain, Mean ± SD | 1.38 ± 2.76 | 0.36 ± 1.36 | 0.89 ± 2.02 | 1.43 ± 2.59 | 3.16 ± 3.93 | <.001 |
| History of falls, n(%) |  |  |  |  |  | <.001 |
| No | 1635 (82.20) | 451 (88.43) | 448 (82.05) | 431 (85.86) | 305 (70.77) |  |
| Yes | 354 (17.80) | 59 (11.57) | 98 (17.95) | 71 (14.14) | 126 (29.23) |  |
| Cognition score, Mean ± SD | 10.84 ± 4.16 | 12.51 ± 3.65 | 11.38 ± 3.73 | 10.15 ± 4.30 | 8.98 ± 4.18 | <.001 |
| Chronic diseases count, Mean ± SD | 1.49 ± 1.38 | 1.13 ± 1.20 | 1.48 ± 1.33 | 1.43 ± 1.33 | 2.00 ± 1.57 | <.001 |

**Note:** Data are based on the unimputed dataset (N = 1,989) to assess the robustness of findings. Values are presented as mean ± standard deviation (SD) for continuous variables and frequency (percentage) for categorical variables. P values were derived from one-way ANOVA for continuous variables and Chi-square tests for categorical variables.

**Abbreviations:** **BMI**, Body Mass Index; **SBP**, Systolic Blood Pressure; **DBP**, Diastolic Blood Pressure; **HPCC**, Household Per Capita Consumption expenditure; **ADL**, Activities of Daily Living; **IADL**, Instrumental Activities of Daily Living; **SRH**, Self-Rated Health;

**Group definitions: G1**, normal-stable sleep and low-stable depression; **G2**, short-stable sleep and low-stable depression; **G3**, normal-increasing sleep and moderate-increasing depression; **G4**, short-decreasing sleep and high-increasing depression.

**Table S7.** Cox proportional hazards regression analysis of the association between joint sleep–depression trajectories and the risk of incident chronic diseases and multimorbidity. (unimputed data).

| **Disease / Group** | **Model 1** | | **Model 2** | | **Model 3** | |
| --- | --- | --- | --- | --- | --- | --- |
|  | HR (95% CI) | *P* | HR (95% CI) | *P* | HR (95% CI) | *P* |
| **Hypertension** |  |  |  |  |  |  |
| G1 | Ref | - | Ref | - | Ref | - |
| G2 | 1.06 (0.91, 1.24) | 0.428 | 1.06 (0.91, 1.24) | 0.427 | 1.12 (0.96, 1.32) | 0.147 |
| G3 | 1.25 (1.07, 1.46) | 0.005 | 1.26 (1.08, 1.47) | 0.004 | 1.34 (1.14, 1.58) | <0.001 |
| G4 | 1.53 (1.30, 1.81) | <0.001 | 1.54 (1.30, 1.82) | <0.001 | 1.74 (1.44, 2.11) | <0.001 |
| **Dyslipidemia** |  |  |  |  |  |  |
| G1 | Ref | - | Ref | - | Ref | - |
| G2 | 1.22 (1.04, 1.44) | 0.015 | 1.25 (1.06, 1.47) | 0.008 | 1.23 (1.04, 1.45) | 0.015 |
| G3 | 1.39 (1.18, 1.64) | <0.001 | 1.40 (1.19, 1.65) | <0.001 | 1.38 (1.16, 1.64) | <0.001 |
| G4 | 1.77 (1.49, 2.11) | <0.001 | 1.83 (1.53, 2.18) | <0.001 | 1.79 (1.47, 2.18) | <0.001 |
| **Diabetes** |  |  |  |  |  |  |
| G1 | Ref | - | Ref | - | Ref | - |
| G2 | 1.35 (1.09, 1.67) | 0.006 | 1.38 (1.12, 1.71) | 0.003 | 1.34 (1.08, 1.67) | 0.008 |
| G3 | 1.25 (1.00, 1.57) | 0.048 | 1.28 (1.02, 1.60) | 0.03 | 1.21 (0.96, 1.53) | 0.104 |
| G4 | 1.89 (1.50, 2.37) | <0.001 | 1.98 (1.57, 2.49) | <0.001 | 1.76 (1.36, 2.28) | <0.001 |
| **Heart disease** |  |  |  |  |  |  |
| G1 | Ref | - | Ref | - | Ref | - |
| G2 | 1.45 (1.22, 1.75) | <0.001 | 1.46 (1.21, 1.77) | <0.001 | 1.40 (1.15, 1.70) | <0.001 |
| G3 | 1.48 (1.22, 1.80) | <0.001 | 1.48 (1.22, 1.80) | <0.001 | 1.38 (1.13, 1.69) | 0.002 |
| G4 | 2.06 (1.68, 2.53) | <0.001 | 2.10 (1.70, 2.58) | <0.001 | 1.78 (1.41, 2.24) | <0.001 |
| **Stroke** |  |  |  |  |  |  |
| G1 | Ref | - | Ref | - | Ref | - |
| G2 | 1.50 (1.11, 2.02) | 0.009 | 1.49 (1.10, 2.02) | 0.009 | 1.41 (1.04, 1.92) | 0.028 |
| G3 | 1.89 (1.40, 2.55) | <0.001 | 1.86 (1.37, 2.51) | <0.001 | 1.71 (1.26, 2.34) | <0.001 |
| G4 | 3.01 (2.23, 4.07) | <0.001 | 2.99 (2.20, 4.05) | <0.001 | 2.52 (1.78, 3.51) | <0.001 |
| **Chronic lung disease** |  |  |  |  |  |  |
| G1 | Ref | - | Ref | - | Ref | - |
| G2 | 1.69 (1.35, 2.11) | <0.001 | 1.68 (1.34, 2.11) | <0.001 | 1.49 (1.18, 1.87) | <0.001 |
| G3 | 2.06 (1.65, 2.58) | <0.001 | 2.07 (1.65, 2.58) | <0.001 | 1.77 (1.42, 2.23) | <0.001 |
| G4 | 3.47 (2.76, 4.35) | <0.001 | 3.47 (2.76, 4.36) | <0.001 | 2.40 (1.86, 3.09) | <0.001 |
| **Asthma** |  |  |  |  |  |  |
| G1 | Ref | - | Ref | - | Ref | - |
| G2 | 1.55 (1.07, 2.24) | 0.021 | 1.54 (1.06, 2.23) | 0.023 | 1.33 (0.91, 1.95) | 0.136 |
| G3 | 1.67 (1.15, 2.42) | 0.007 | 1.63 (1.12, 2.37) | 0.01 | 1.38 (0.94, 2.02) | 0.105 |
| G4 | 2.89 (2.00, 4.19) | <0.001 | 2.84 (1.96, 4.12) | <0.001 | 2.09 (1.39, 3.14) | <0.001 |
| **Liver disease** |  |  |  |  |  |  |
| G1 | Ref | - | Ref | - | Ref | - |
| G2 | 1.19 (0.91, 1.57) | 0.219 | 1.20 (0.91, 1.58) | 0.205 | 1.05 (0.79, 1.40) | 0.727 |
| G3 | 1.35 (1.02, 1.78) | 0.036 | 1.36 (1.03, 1.79) | 0.032 | 1.14 (0.85, 1.52) | 0.378 |
| G4 | 1.99 (1.49, 2.65) | <0.001 | 2.02 (1.51, 2.69) | <0.001 | 1.56 (1.21, 2.03) | <0.001 |
| **Cancer** |  |  |  |  |  |  |
| G1 | Ref | - | Ref | - | Ref | - |
| G2 | 1.33 (0.83, 2.13) | 0.229 | 1.28 (0.80, 2.05) | 0.307 | 1.14 (0.71, 1.85) | 0.586 |
| G3 | 1.51 (0.94, 2.43) | 0.09 | 1.48 (0.92, 2.39) | 0.105 | 1.28 (0.78, 2.10) | 0.326 |
| G4 | 2.01 (1.23, 3.29) | 0.006 | 1.86 (1.13, 3.05) | 0.014 | 1.37 (1.03, 1.70) | 0.035 |
| **Digestive disease** |  |  |  |  |  |  |
| G1 | Ref | - | Ref | - | Ref | - |
| G2 | 1.35 (1.13, 1.61) | 0.001 | 1.35 (1.13, 1.62) | 0.001 | 1.19 (0.99, 1.43) | 0.059 |
| G3 | 1.41 (1.18, 1.69) | <0.001 | 1.42 (1.19, 1.71) | <0.001 | 1.21 (1.00, 1.46) | 0.052 |
| G4 | 2.13 (1.76, 2.59) | <0.001 | 2.15 (1.77, 2.61) | <0.001 | 1.54 (1.24, 1.91) | <0.001 |
| **Kidney disease** |  |  |  |  |  |  |
| G1 | Ref | - | Ref | - | Ref | - |
| G2 | 2.03 (1.57, 2.62) | <0.001 | 2.03 (1.57, 2.63) | <0.001 | 1.78 (1.37, 2.31) | <0.001 |
| G3 | 1.84 (1.41, 2.41) | <0.001 | 1.85 (1.41, 2.43) | <0.001 | 1.51 (1.14, 1.99) | 0.004 |
| G4 | 3.56 (2.72, 4.65) | <0.001 | 3.58 (2.74, 4.69) | <0.001 | 2.31 (1.71, 3.13) | <0.001 |
| **Arthritis** |  |  |  |  |  |  |
| G1 | Ref | - | Ref | - | Ref | - |
| G2 | 1.62 (1.37, 1.90) | <0.001 | 1.61 (1.37, 1.89) | <0.001 | 1.47 (1.24, 1.73) | <0.001 |
| G3 | 1.76 (1.49, 2.08) | <0.001 | 1.76 (1.49, 2.07) | <0.001 | 1.51 (1.27, 1.80) | <0.001 |
| G4 | 2.37 (1.96, 2.86) | <0.001 | 2.34 (1.94, 2.82) | <0.001 | 1.72 (1.40, 2.12) | <0.001 |
| **Memory-related disorders** |  |  |  |  |  |  |
| G1 | Ref | - | Ref | - | Ref | - |
| G2 | 1.97 (1.34, 2.90) | <0.001 | 1.96 (1.33, 2.88) | <0.001 | 1.68 (1.13, 2.49) | 0.01 |
| G3 | 2.75 (1.88, 4.01) | <0.001 | 2.77 (1.90, 4.05) | <0.001 | 2.26 (1.53, 3.35) | <0.001 |
| G4 | 5.04 (3.47, 7.33) | <0.001 | 5.04 (3.46, 7.34) | <0.001 | 3.52 (2.34, 5.32) | <0.001 |
| **Multimorbidity** |  |  |  |  |  |  |
| G1 | Ref | - | Ref | - | Ref | - |
| G2 | 1.44 (1.28, 1.63) | <0.001 | 1.45 (1.28, 1.63) | <0.001 | 1.36 (1.20, 1.54) | <0.001 |
| G3 | 1.65 (1.46, 1.86) | <0.001 | 1.66 (1.47, 1.87) | <0.001 | 1.51 (1.33, 1.72) | <0.001 |
| G4 | 2.52 (2.19, 2.91) | <0.001 | 2.54 (2.20, 2.93) | <0.001 | 2.18 (1.86, 2.55) | <0.001 |

**Note:** Data are based on the unimputed data to assess the robustness of the primary findings. Results are presented as Hazard Ratio (95% Confidence Interval).

Model 1: Adjusted for age, sex, educational attainment, marital status, Hukou status, residence, and geographic region.

Model 2: Additionally adjusted for smoking status, alcohol consumption, nap duration, social participation, and social isolation.

Model 3: Further adjusted for body mass index (BMI), waist circumference, blood pressure, grip strength, balance, chair stand test, lung function, ADL/IADL disability, sensory function, tooth loss, self-rated health, body pain, history of falls, cognitive function, and baseline chronic disease count.

**Group definitions: G1**, normal-stable sleep and low-stable depression; **G2**, short-stable sleep and low-stable depression; **G3**, normal-increasing sleep and moderate-increasing depression; **G4**, short-decreasing sleep and high-increasing depression.

**Abbreviations: HR**, Hazard Ratio; **CI**, Confidence Interval; **Ref**, Reference group.

**Table S8.** Comparative performance metrics of seven machine learning algorithms (unimputed data).

| **Model** | **AUC** | **Accuracy** | **Precision** | **Sensitivity** | **Specificity** | **F1 Score** |
| --- | --- | --- | --- | --- | --- | --- |
| Logistic | 0.7921 | 0.8323 | 0.6176 | 0.2809 | 0.9598 | 0.4062 |
| Decision Tree | 0.7372 | 0.8204 | 0.5546 | 0.2207 | 0.9590 | 0.3458 |
| Random Forest | 0.778 | 0.8285 | 0.5855 | 0.2977 | 0.9513 | 0.4147 |
| XGBoost | 0.7960 | 0.8348 | 0.6429 | 0.2709 | 0.9652 | 0.4212 |
| LightGBM | 0.7795 | 0.8222 | 0.5494 | 0.2977 | 0.9435 | 0.4061 |
| SVM | 0.7905 | 0.8279 | 0.6087 | 0.2341 | 0.9652 | 0.3582 |
| ANN | 0.7915 | 0.8247 | 0.5521 | 0.3545 | 0.9335 | 0.4318 |

**Abbreviations: AUC**, Area Under the Receiver Operating Characteristic Curve; **SVM**, Support Vector Machine; **ANN**, Artificial Neural Network; **XGBoost**, eXtreme Gradient Boosting; **LightGBM**, Light Gradient Boosting Machine.

**Table S9.** Cox proportional hazards regression analysis of the association between joint sleep–depression trajectories and the risk of incident chronic diseases and multimorbidity during the 2018–2020 follow-up period.

| **Disease / Group** | **Model 1** | | **Model 2** | | **Model 3** | |
| --- | --- | --- | --- | --- | --- | --- |
|  | HR (95% CI) | *P* | HR (95% CI) | *P* | HR (95% CI) | *P* |
| **Hypertension** |  |  |  |  |  |  |
| G1 | Ref | - | Ref | - | Ref | - |
| G2 | 0.85 (0.66, 1.10) | 0.208 | 0.85 (0.66, 1.10) | 0.213 | 0.90 (0.69, 1.16) | 0.398 |
| G3 | 0.95 (0.74, 1.22) | 0.684 | 0.96 (0.75, 1.23) | 0.754 | 1.01 (0.77, 1.29) | 0.879 |
| G4 | 1.36 (1.05, 1.76) | 0.018 | 1.37 (1.06, 1.78) | 0.016 | 1.43 (1.07, 1.90) | 0.015 |
| **Dyslipidemia** |  |  |  |  |  |  |
| G1 | Ref | - | Ref | - | Ref | - |
| G2 | 1.26 (1.00, 1.58) | 0.051 | 1.29 (1.03, 1.63) | 0.03 | 1.29 (1.02, 1.63) | 0.031 |
| G3 | 1.48 (1.18, 1.86) | <0.001 | 1.49 (1.19, 1.87) | <0.001 | 1.48 (1.18, 1.87) | <0.001 |
| G4 | 1.72 (1.35, 2.20) | <0.001 | 1.79 (1.40, 2.28) | <0.001 | 1.82 (1.40, 2.38) | <0.001 |
| **Diabetes** |  |  |  |  |  |  |
| G1 | Ref | - | Ref | - | Ref | - |
| G2 | 1.23 (0.90, 1.68) | 0.19 | 1.24 (0.91, 1.69) | 0.18 | 1.19 (0.87, 1.64) | 0.271 |
| G3 | 1.30 (0.95, 1.77) | 0.102 | 1.29 (0.95, 1.77) | 0.108 | 1.23 (0.89, 1.70) | 0.204 |
| G4 | 1.87 (1.36, 2.58) | <0.001 | 1.89 (1.37, 2.60) | <0.001 | 1.68 (1.18, 2.39) | 0.004 |
| **Heart disease** |  |  |  |  |  |  |
| G1 | Ref | - | Ref | - | Ref | - |
| G2 | 1.29 (0.98, 1.71) | 0.075 | 1.32 (1.00, 1.75) | 0.053 | 1.24 (0.93, 1.65) | 0.14 |
| G3 | 1.19 (0.89, 1.59) | 0.241 | 1.19 (0.89, 1.59) | 0.249 | 1.07 (0.80, 1.45) | 0.648 |
| G4 | 1.70 (1.26, 2.29) | 0.001 | 1.74 (1.29, 2.35) | <0.001 | 1.45 (1.04, 2.01) | 0.027 |
| **Stroke** |  |  |  |  |  |  |
| G1 | Ref | - | Ref | - | Ref | - |
| G2 | 1.79 (1.09, 2.94) | 0.022 | 1.73 (1.06, 2.85) | 0.03 | 1.67 (1.01, 2.76) | 0.044 |
| G3 | 2.38 (1.47, 3.86) | <0.001 | 2.34 (1.44, 3.79) | <0.001 | 2.19 (1.34, 3.59) | 0.002 |
| G4 | 4.46 (2.78, 7.15) | <0.001 | 4.24 (2.63, 6.81) | <0.001 | 3.65 (2.20, 6.06) | <0.001 |
| **Chronic lung disease** |  |  |  |  |  |  |
| G1 | Ref | - | Ref | - | Ref | - |
| G2 | 1.79 (1.24, 2.59) | 0.002 | 1.76 (1.22, 2.53) | 0.003 | 1.63 (1.13, 2.37) | 0.01 |
| G3 | 1.66 (1.13, 2.43) | 0.009 | 1.66 (1.13, 2.43) | 0.01 | 1.50 (1.02, 2.22) | 0.041 |
| G4 | 2.79 (1.90, 4.10) | <0.001 | 2.70 (1.83, 3.97) | <0.001 | 2.17 (1.43, 3.29) | <0.001 |
| **Asthma** |  |  |  |  |  |  |
| G1 | Ref | - | Ref | - | Ref | - |
| G2 | 1.63 (0.98, 2.73) | 0.062 | 1.63 (0.97, 2.72) | 0.063 | 1.45 (0.86, 2.45) | 0.161 |
| G3 | 1.45 (0.85, 2.47) | 0.176 | 1.41 (0.83, 2.42) | 0.204 | 1.20 (0.70, 2.08) | 0.507 |
| G4 | 2.29 (1.35, 3.88) | 0.002 | 2.25 (1.32, 3.84) | 0.003 | 1.71 (1.10, 2.97) | 0.018 |
| **Liver disease** |  |  |  |  |  |  |
| G1 | Ref | - | Ref | - | Ref | - |
| G2 | 1.11 (0.71, 1.75) | 0.65 | 1.11 (0.71, 1.75) | 0.646 | 1.02 (0.64, 1.61) | 0.941 |
| G3 | 1.07 (0.67, 1.72) | 0.771 | 1.08 (0.68, 1.74) | 0.743 | 0.96 (0.59, 1.55) | 0.852 |
| G4 | 1.76 (1.11, 2.80) | 0.016 | 1.75 (1.10, 2.79) | 0.019 | 1.47 (1.01, 2.18) | 0.036 |
| **Cancer** |  |  |  |  |  |  |
| G1 | Ref | - | Ref | - | Ref | - |
| G2 | 1.40 (0.75, 2.63) | 0.294 | 1.39 (0.74, 2.61) | 0.309 | 1.17 (0.61, 2.22) | 0.638 |
| G3 | 1.43 (0.76, 2.72) | 0.271 | 1.43 (0.75, 2.73) | 0.273 | 1.13 (0.58, 2.19) | 0.719 |
| G4 | 1.41 (0.69, 2.86) | 0.347 | 1.39 (0.68, 2.84) | 0.371 | 0.90 (0.41, 1.97) | 0.798 |
| **Digestive disease** |  |  |  |  |  |  |
| G1 | Ref | - | Ref | - | Ref | - |
| G2 | 1.25 (0.94, 1.67) | 0.131 | 1.26 (0.94, 1.68) | 0.127 | 1.13 (0.84, 1.52) | 0.411 |
| G3 | 1.26 (0.94, 1.69) | 0.122 | 1.26 (0.94, 1.69) | 0.124 | 1.09 (0.80, 1.47) | 0.588 |
| G4 | 2.03 (1.49, 2.77) | <0.001 | 2.04 (1.49, 2.79) | <0.001 | 1.51 (1.07, 2.11) | 0.018 |
| **Kidney disease** |  |  |  |  |  |  |
| G1 | Ref | - | Ref | - | Ref | - |
| G2 | 1.52 (1.03, 2.24) | 0.035 | 1.50 (1.02, 2.22) | 0.04 | 1.33 (0.90, 1.97) | 0.159 |
| G3 | 2.00 (1.37, 2.92) | <0.001 | 1.96 (1.34, 2.88) | <0.001 | 1.60 (1.08, 2.37) | 0.019 |
| G4 | 3.15 (2.13, 4.66) | <0.001 | 3.04 (2.05, 4.51) | <0.001 | 1.97 (1.28, 3.03) | 0.002 |
| **Arthritis** |  |  |  |  |  |  |
| G1 | Ref | - | Ref | - | Ref | - |
| G2 | 1.02 (0.76, 1.37) | 0.897 | 1.00 (0.74, 1.34) | 0.982 | 0.96 (0.71, 1.29) | 0.785 |
| G3 | 1.21 (0.91, 1.61) | 0.188 | 1.21 (0.91, 1.60) | 0.199 | 1.13 (0.84, 1.51) | 0.433 |
| G4 | 1.43 (1.12, 1.95) | <0.001 | 1.39 (1.10, 1.90) | 0.007 | 1.21 (1.01, 1.54) | 0.047 |
| **Memory-related disorders** |  |  |  |  |  |  |
| G1 | Ref | - | Ref | - | Ref | - |
| G2 | 2.17 (1.45, 3.24) | <0.001 | 2.13 (1.42, 3.19) | <0.001 | 1.99 (1.32, 2.98) | 0.001 |
| G3 | 2.91 (1.96, 4.34) | <0.001 | 2.89 (1.94, 4.31) | <0.001 | 2.60 (1.73, 3.91) | <0.001 |
| G4 | 4.67 (3.14, 6.95) | <0.001 | 4.51 (3.02, 6.73) | <0.001 | 3.57 (2.33, 5.46) | <0.001 |
| **Multimorbidity** |  |  |  |  |  |  |
| G1 | Ref | - | Ref | - | Ref | - |
| G2 | 1.13 (0.92, 1.39) | 0.257 | 1.11 (0.90, 1.37) | 0.321 | 1.09 (0.88, 1.34) | 0.437 |
| G3 | 1.30 (1.05, 1.60) | 0.015 | 1.30 (1.05, 1.60) | 0.014 | 1.25 (1.01, 1.55) | 0.041 |
| G4 | 1.70 (1.31, 2.19) | <0.001 | 1.67 (1.29, 2.17) | <0.001 | 1.51 (1.15, 2.00) | 0.003 |

**Note:** Data are derived from the landmark sensitivity analysis restricted to the 2018–2020 follow-up period. Results are presented as Hazard Ratio (95% Confidence Interval).

Model 1: Adjusted for age, sex, educational attainment, marital status, Hukou status, residence, and geographic region.

Model 2: Additionally adjusted for smoking status, alcohol consumption, nap duration, social participation, and social isolation.

Model 3: Further adjusted for body mass index (BMI), waist circumference, blood pressure, grip strength, balance, chair stand test, lung function, ADL/IADL disability, sensory function, tooth loss, self-rated health, body pain, history of falls, cognitive function, and baseline chronic disease count.

**Group definitions: G1**, normal-stable sleep and low-stable depression; **G2**, short-stable sleep and low-stable depression; **G3**, normal-increasing sleep and moderate-increasing depression; **G4**, short-decreasing sleep and high-increasing depression.

**Abbreviations: HR**, Hazard Ratio; **CI**, Confidence Interval; **Ref**, Reference group.

**Table S10.** Cox proportional hazards regression analysis comparing the risk of incident chronic diseases and multimorbidity between Group 2 and Group 3.

| **Disease / Group** | **Model 1** | | **Model 2** | | **Model 3** | |
| --- | --- | --- | --- | --- | --- | --- |
|  | HR (95% CI) | *P* | HR (95% CI) | *P* | HR (95% CI) | *P* |
| **Hypertension** |  |  |  |  |  |  |
| G2 | Ref | - | Ref | - | Ref | - |
| G3 | 1.11 (0.99, 1.26) | 0.082 | 1.12 (0.99, 1.26) | 0.071 | 1.12 (0.99, 1.26) | 0.076 |
| **Dyslipidemia** |  |  |  |  |  |  |
| G2 | Ref | - | Ref | - | Ref | - |
| G3 | 1.12 (0.99, 1.26) | 0.078 | 1.10 (0.97, 1.24) | 0.122 | 1.09 (0.96, 1.23) | 0.181 |
| **Diabetes** |  |  |  |  |  |  |
| G2 | Ref | - | Ref | - | Ref | - |
| G3 | 0.98 (0.83, 1.15) | 0.821 | 0.97 (0.82, 1.14) | 0.692 | 0.93 (0.79, 1.10) | 0.375 |
| **Cancer** |  |  |  |  |  |  |
| G2 | Ref | - | Ref | - | Ref | - |
| G3 | 0.96 (0.67, 1.38) | 0.83 | 0.99 (0.69, 1.42) | 0.95 | 0.95 (0.66, 1.38) | 0.8 |
| **Chronic lung disease** |  |  |  |  |  |  |
| G2 | Ref | - | Ref | - | Ref | - |
| G3 | 1.16 (0.99, 1.35) | 0.064 | 1.16 (0.99, 1.35) | 0.071 | 1.12 (0.96, 1.31) | 0.147 |
| **Liver disease** |  |  |  |  |  |  |
| G2 | Ref | - | Ref | - | Ref | - |
| G3 | 1.12 (0.90, 1.39) | 0.312 | 1.12 (0.91, 1.40) | 0.290 | 1.08 (0.87, 1.34) | 0.486 |
| **Heart disease** |  |  |  |  |  |  |
| G2 | Ref | - | Ref | - | Ref | - |
| G3 | 1.00 (0.86, 1.15) | 0.958 | 0.99 (0.86, 1.14) | 0.861 | 0.95 (0.83, 1.10) | 0.522 |
| **Stroke** |  |  |  |  |  |  |
| G2 | Ref | - | Ref | - | Ref | - |
| G3 | 1.35 (1.09, 1.66) | 0.007 | 1.34 (1.09, 1.65) | 0.009 | 1.31 (1.06, 1.61) | 0.012 |
| **Kidney disease** |  |  |  |  |  |  |
| G2 | Ref | - | Ref | - | Ref | - |
| G3 | 0.98 (0.82, 1.18) | 0.862 | 1.00 (0.83, 1.19) | 0.967 | 0.92 (0.77, 1.10) | 0.351 |
| **Digestive disease** |  |  |  |  |  |  |
| G2 | Ref | - | Ref | - | Ref | - |
| G3 | 1.04 (0.91, 1.19) | 0.57 | 1.04 (0.91, 1.19) | 0.53 | 1.00 (0.87, 1.14) | 0.96 |
| **Memory-related disorders** |  |  |  |  |  |  |
| G2 | Ref | - | Ref | - | Ref | - |
| G3 | 1.29 (1.01, 1.64) | 0.036 | 1.31 (1.03, 1.67) | 0.031 | 1.23 (1.01, 1.58) | 0.045 |
| **Arthritis** |  |  |  |  |  |  |
| G2 | Ref | - | Ref | - | Ref | - |
| G3 | 1.02 (0.90, 1.15) | 0.782 | 1.02 (0.90, 1.16) | 0.717 | 0.96 (0.85, 1.09) | 0.542 |
| **Asthma** |  |  |  |  |  |  |
| G2 | Ref | - | Ref | - | Ref | - |
| G3 | 1.07 (0.82, 1.40) | 0.592 | 1.06 (0.81, 1.39) | 0.661 | 1.05 (0.80, 1.37) | 0.742 |
| **Multimorbidity** |  |  |  |  |  |  |
| G2 | Ref | - | Ref | - | Ref | - |
| G3 | 1.07 (0.97, 1.19) | 0.152 | 1.08 (0.97, 1.19) | 0.151 | 1.05 (0.95, 1.16) | 0.376 |

**Note:** Data are presented as Hazard Ratio (95% Confidence Interval).

Model 1: Adjusted for age, sex, educational attainment, marital status, Hukou status, residence, and geographic region.

Model 2: Additionally adjusted for smoking status, alcohol consumption, nap duration, social participation, and social isolation.

Model 3: Further adjusted for body mass index (BMI), waist circumference, blood pressure, grip strength, balance, chair stand test, lung function, ADL/IADL disability, sensory function, tooth loss, self-rated health, body pain, history of falls, cognitive function, and baseline chronic disease count.

**Group definitions: G2**, short-stable sleep and low-stable depression; **G3**, normal-increasing sleep and moderate-increasing depression.

**Abbreviations: HR**, Hazard Ratio; **CI**, Confidence Interval; **Ref**, Reference group.


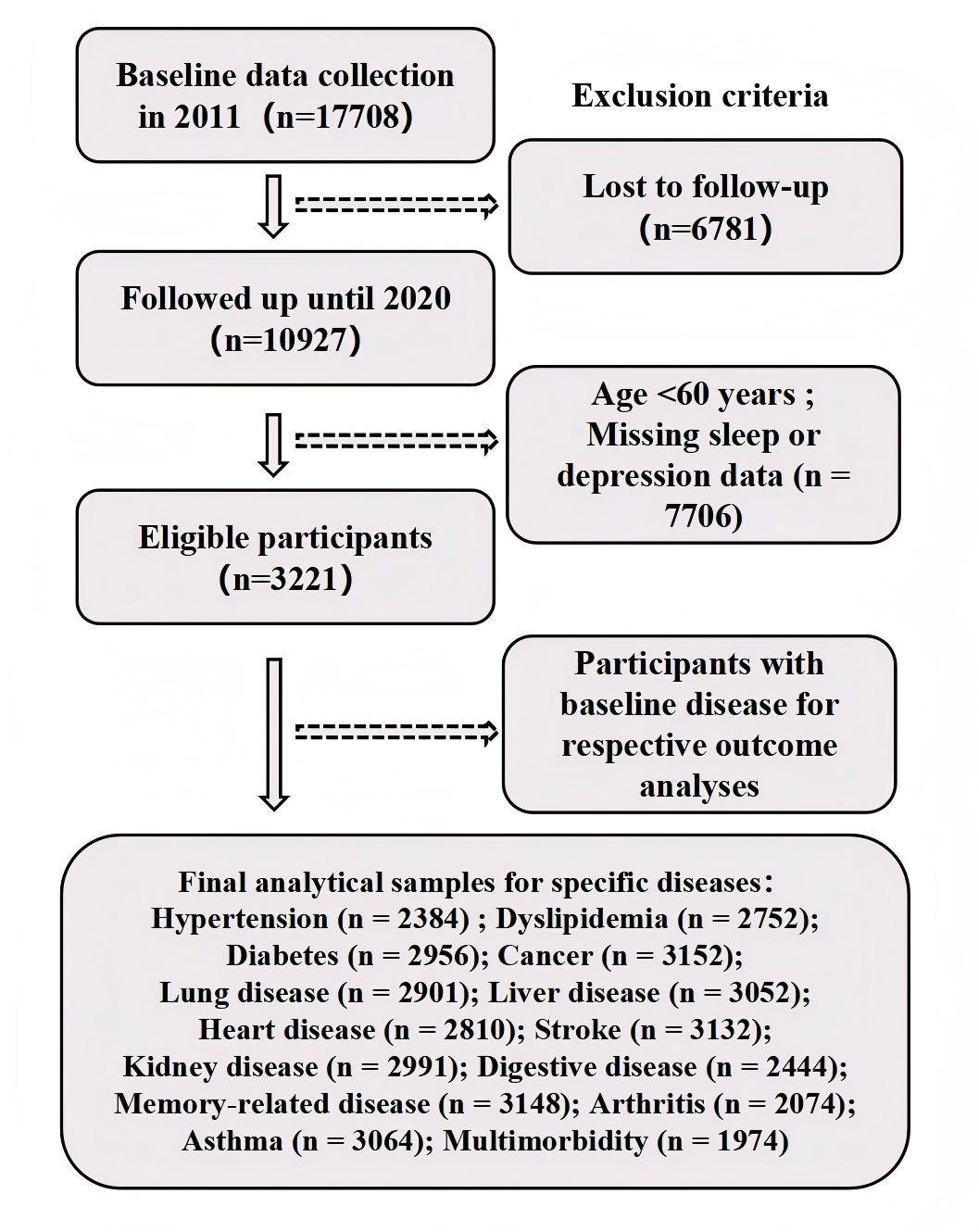


**Figure S1.** Flow diagram for participants included in the study.


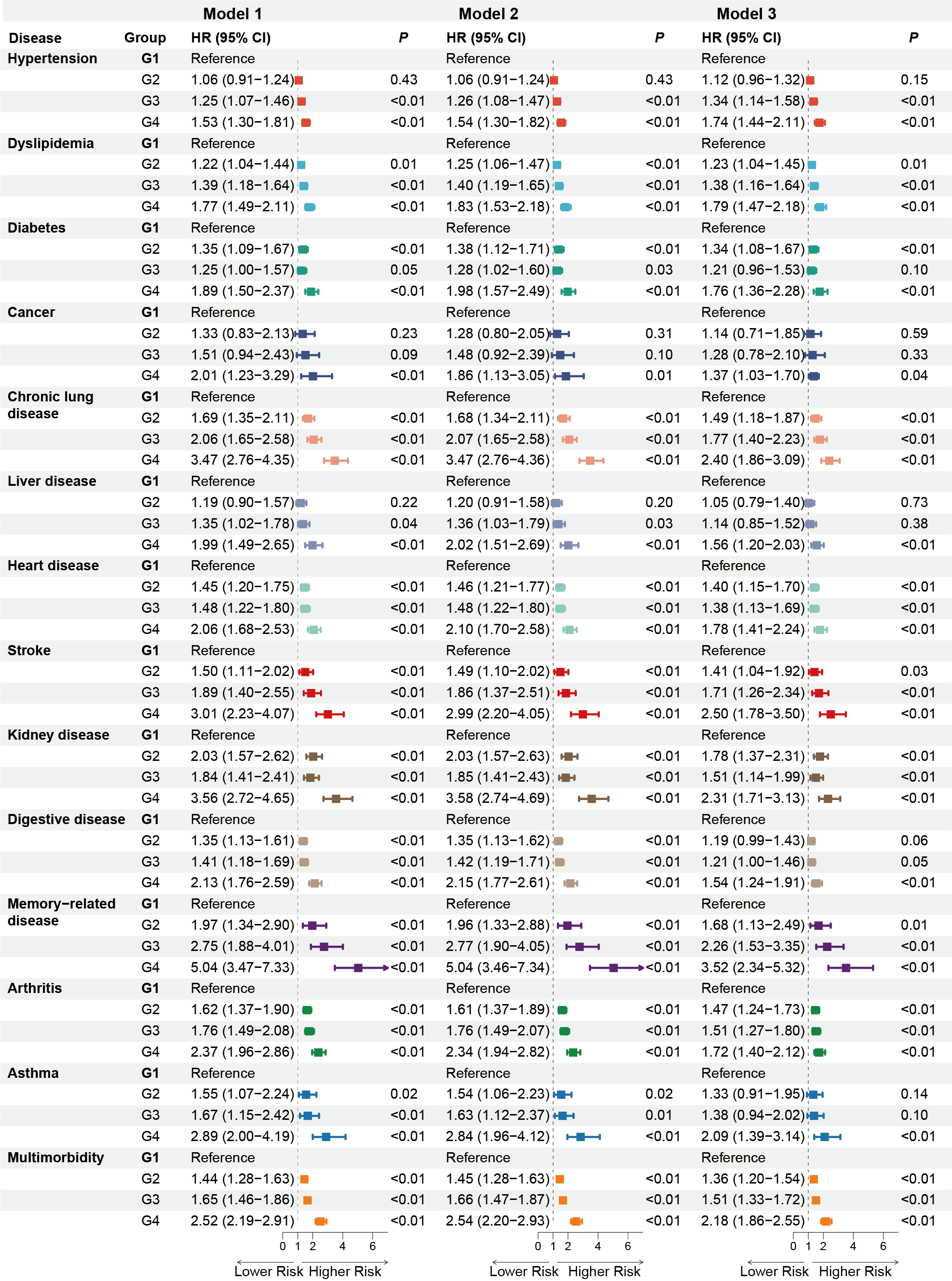


**Figure S2.** Forest plot of joint sleep–depression trajectories and risk of multiple incident chronic diseases and multimorbidity (unimputed data).


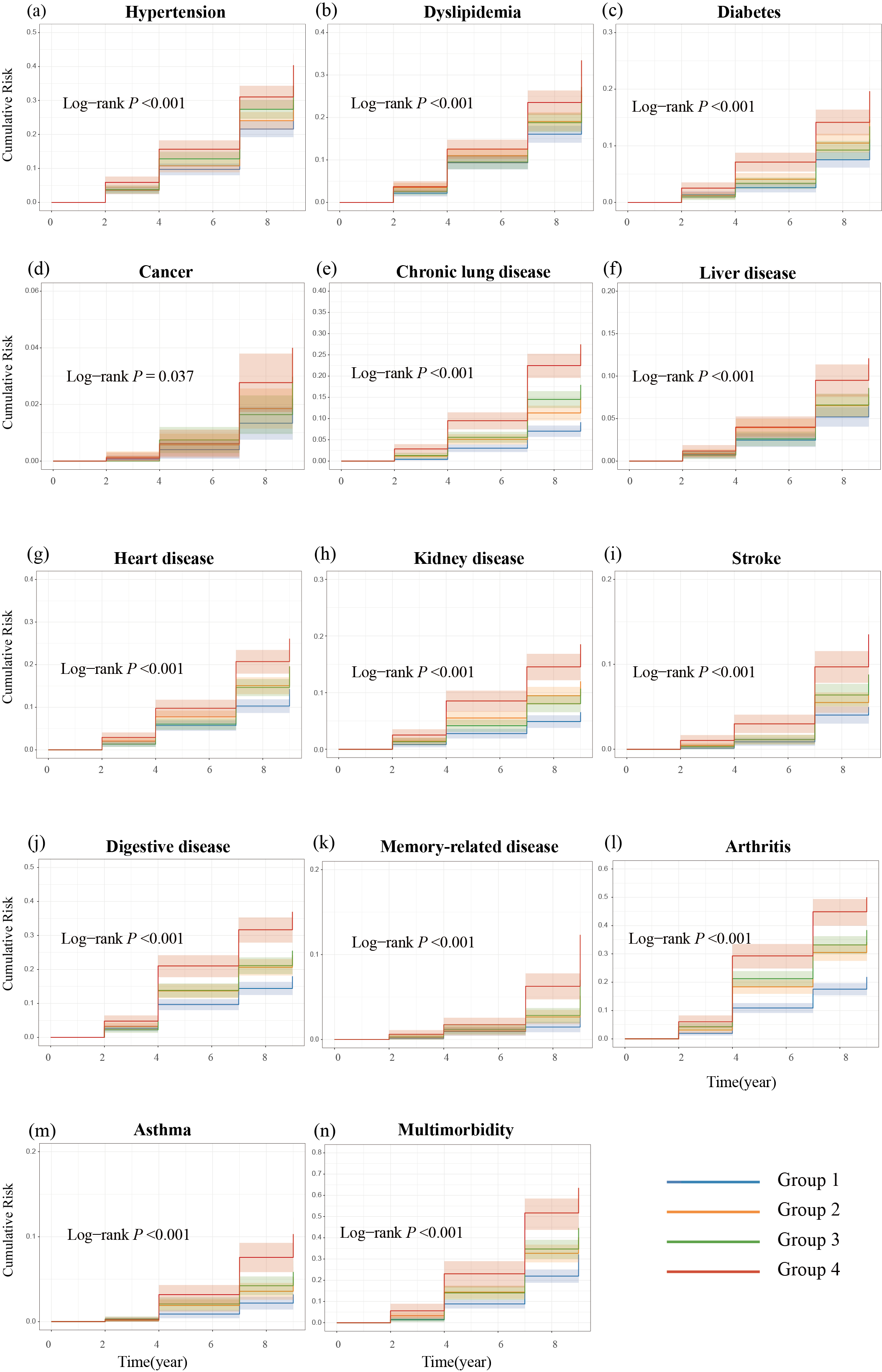


**Figure S3.** Kaplan–Meier cumulative incidence curves for chronic diseases and multimorbidity stratified by joint trajectory groups(unimputed data). (a–m) Cumulative incidence curves for the 13 specific chronic diseases; (n) cumulative incidence curve for multimorbidity.


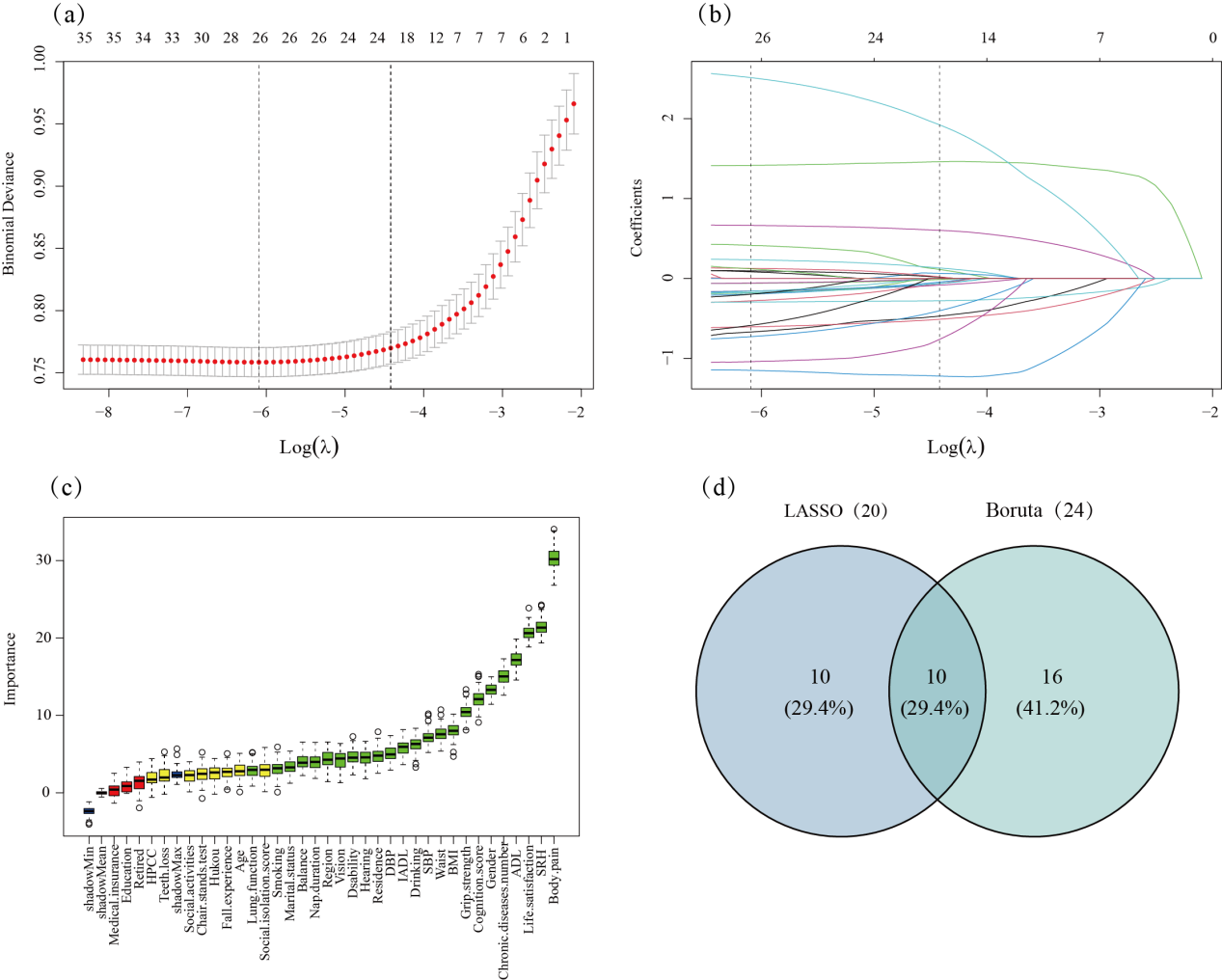


**Figure S4.** Feature selection results based on unimputed data. (a) LASSO parameter selection via cross-validation; (b) LASSO coefficient profiles: The left dashed line indicates the minimum error(λmin), while the right dashed line indicates the 1-standard-error rule(λ1se); (c) Feature importance ranking via the Boruta algorithm: green boxplots indicate confirmed important features, and blue boxplots represent shadow features; (d) Venn diagram illustrating the intersection of selected features.


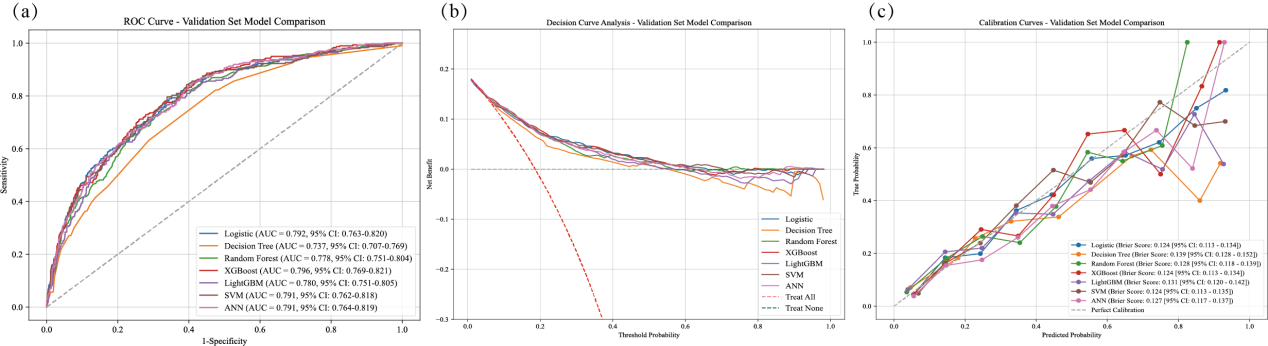


**Figure S5.** Comparative performance of seven predictive models using unimputed data. (a) ROC curves; (b) decision curve analysis (DCA); (c) calibration curves.


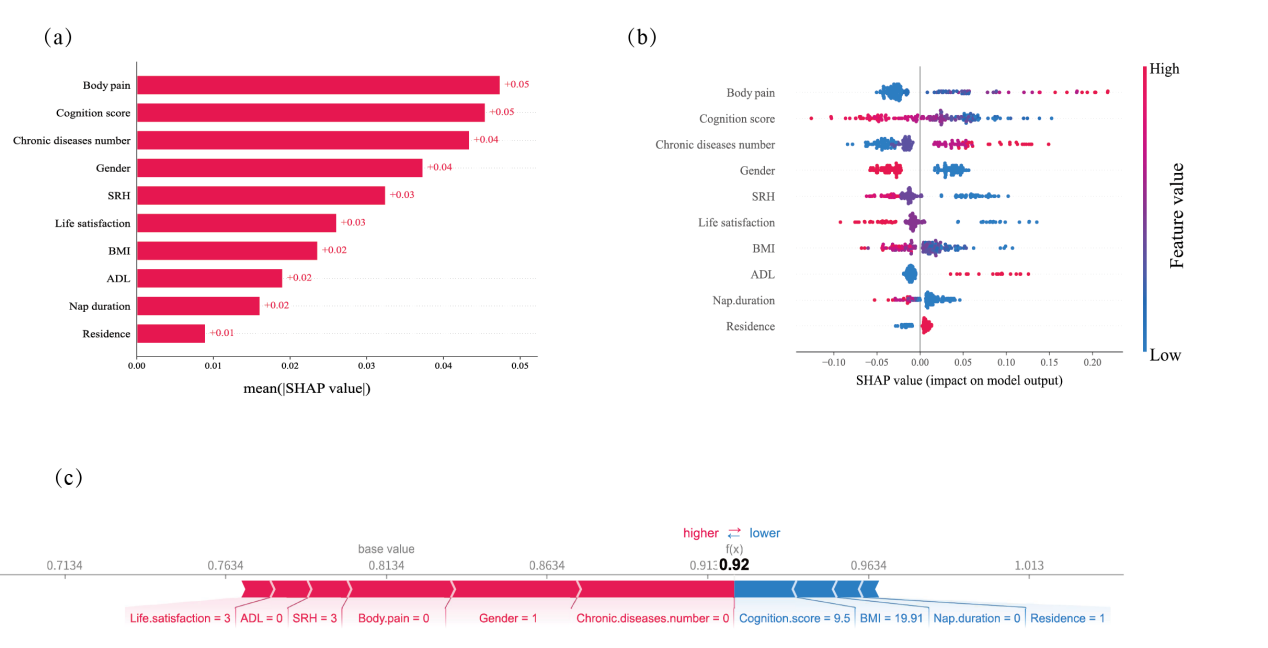


**Figure S6.** SHAP-based interpretability for the top-performing model (unimputed data). (a) Bar plot ranking features by mean absolute SHAP value; (b) SHAP beeswarm plot illustrating global feature importance and directional effects (red and blue dots indicate high and low feature values, respectively); (c) local interpretability analysis via a force plot for a representative individual case.


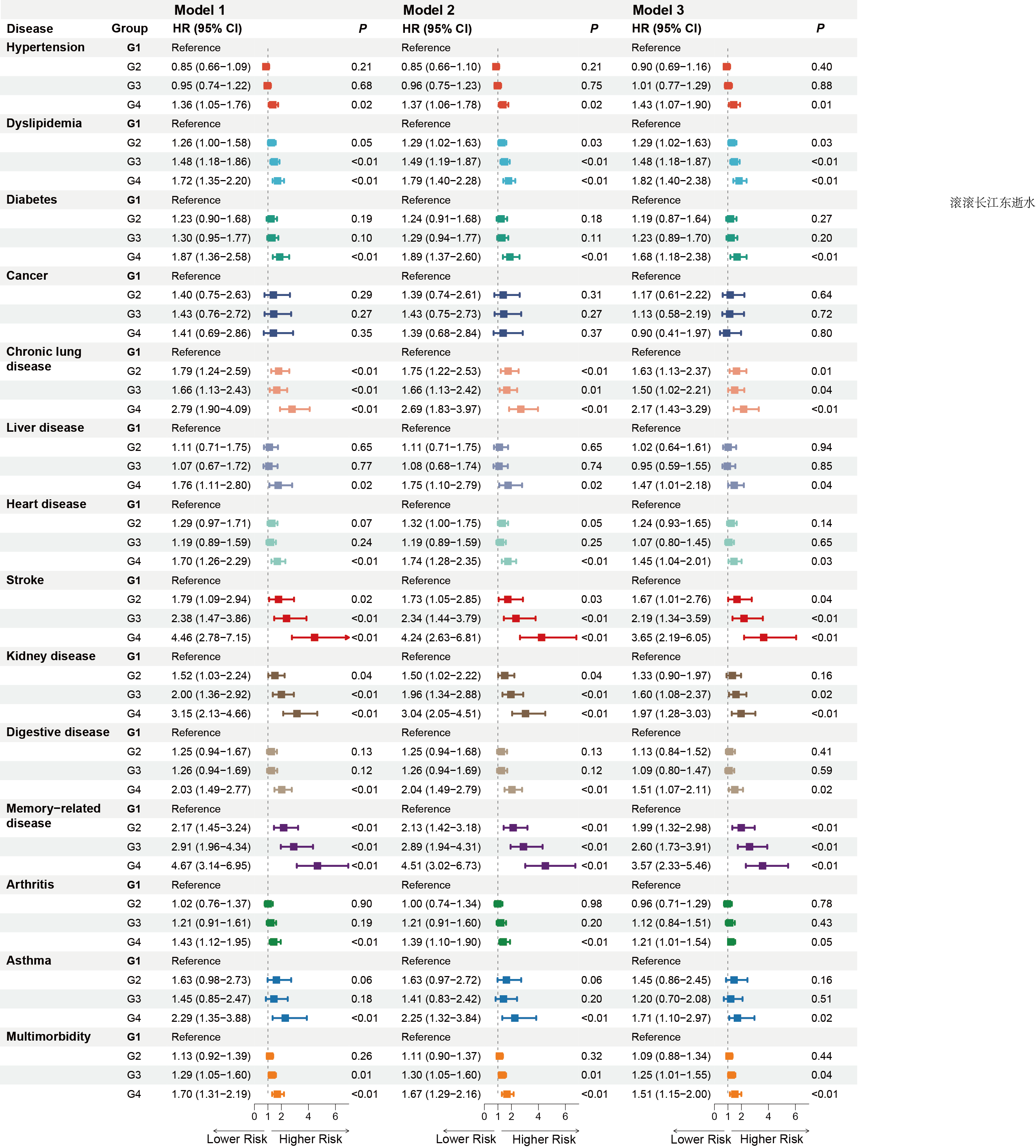


**Figure S7.** Forest plot of joint sleep–depression trajectories and risk of multiple incident chronic diseases and multimorbidity during the 2018–2020 follow-up period.


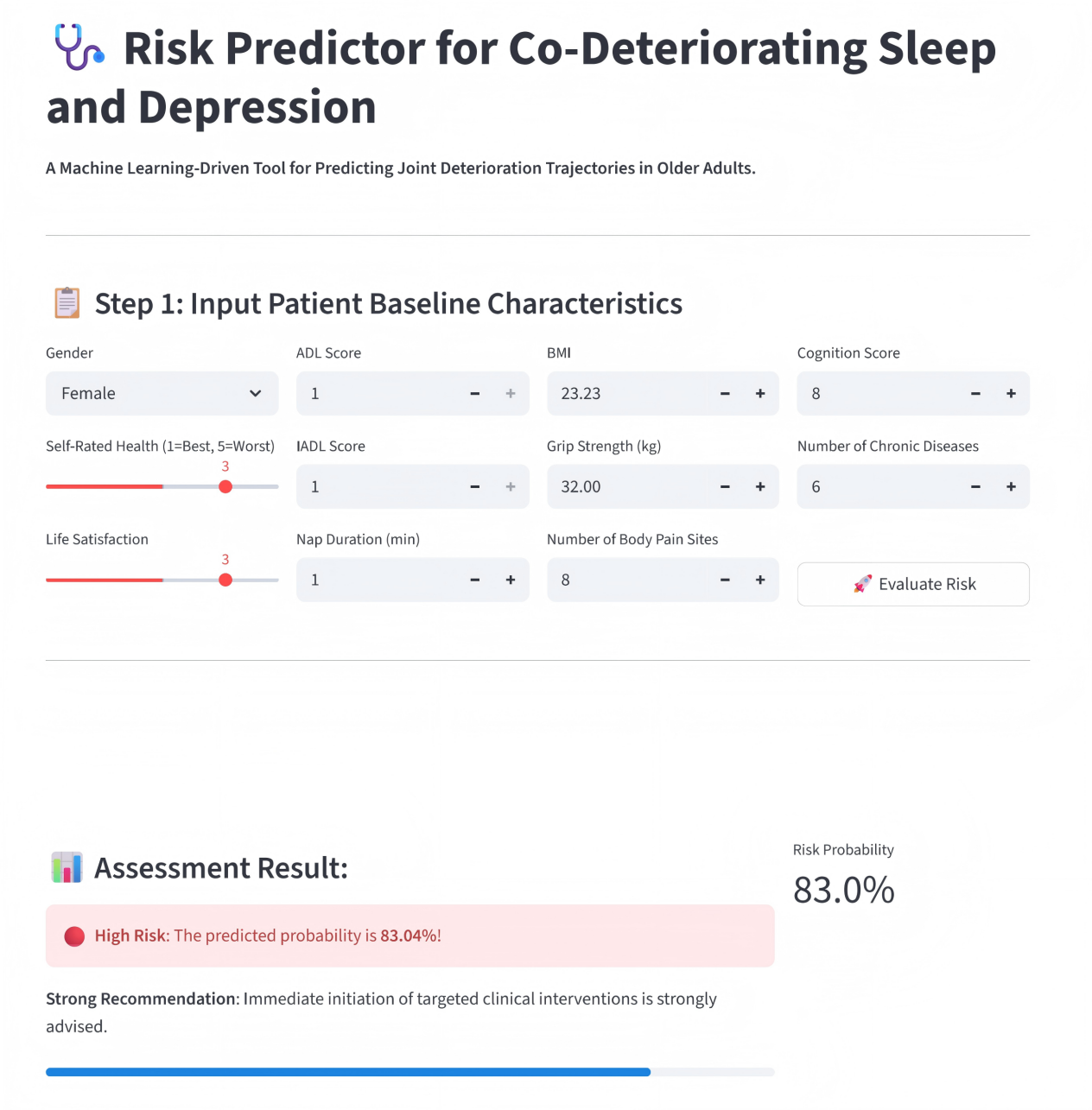


**Figure S8.** Web-based risk calculator for predicting membership in the high-risk joint sleep–depression trajectory.
